# Supplementary material for: Empowering Future Physicians: Enhancing Naloxone Competency Through Early Harm Reduction Training in Medical Education
Source: MedEdPORTAL. 2025 Feb 14;21:11499. doi: 10.15766/mep_2374-8265.11499 (PMC11825861; doi:10.15766/mep_2374-8265.11499)
Supplement: Supplementary file 1 — Facilitator Guide.docxOpioid Overdose Statistics Lecture.pptxHarm Reduction Initiatives Lecture.pptxCase-Based Discussion Scenario.pptxOSCE-Style Checklist.docxTraining Session Confidence Survey.docx [file mep_2374-8265.11499-s001.zip › B. Opioid Overdose Statistics Lecture.pptx]

## Slide 1
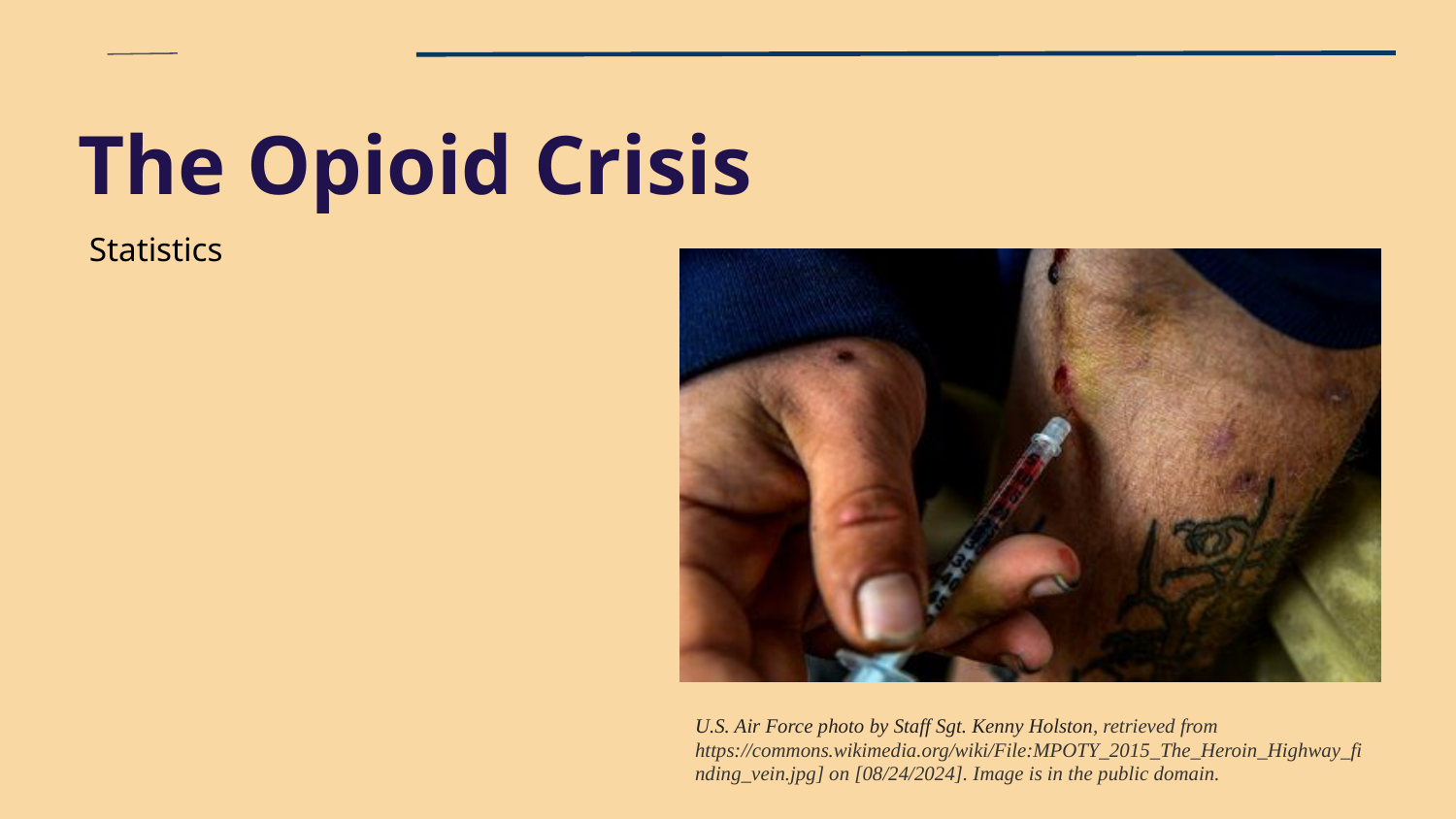

The Opioid Crisis
Statistics
U.S. Air Force photo by Staff Sgt. Kenny Holston, retrieved from https://commons.wikimedia.org/wiki/File:MPOTY_2015_The_Heroin_Highway_finding_vein.jpg] on [08/24/2024]. Image is in the public domain.

## Slide 2
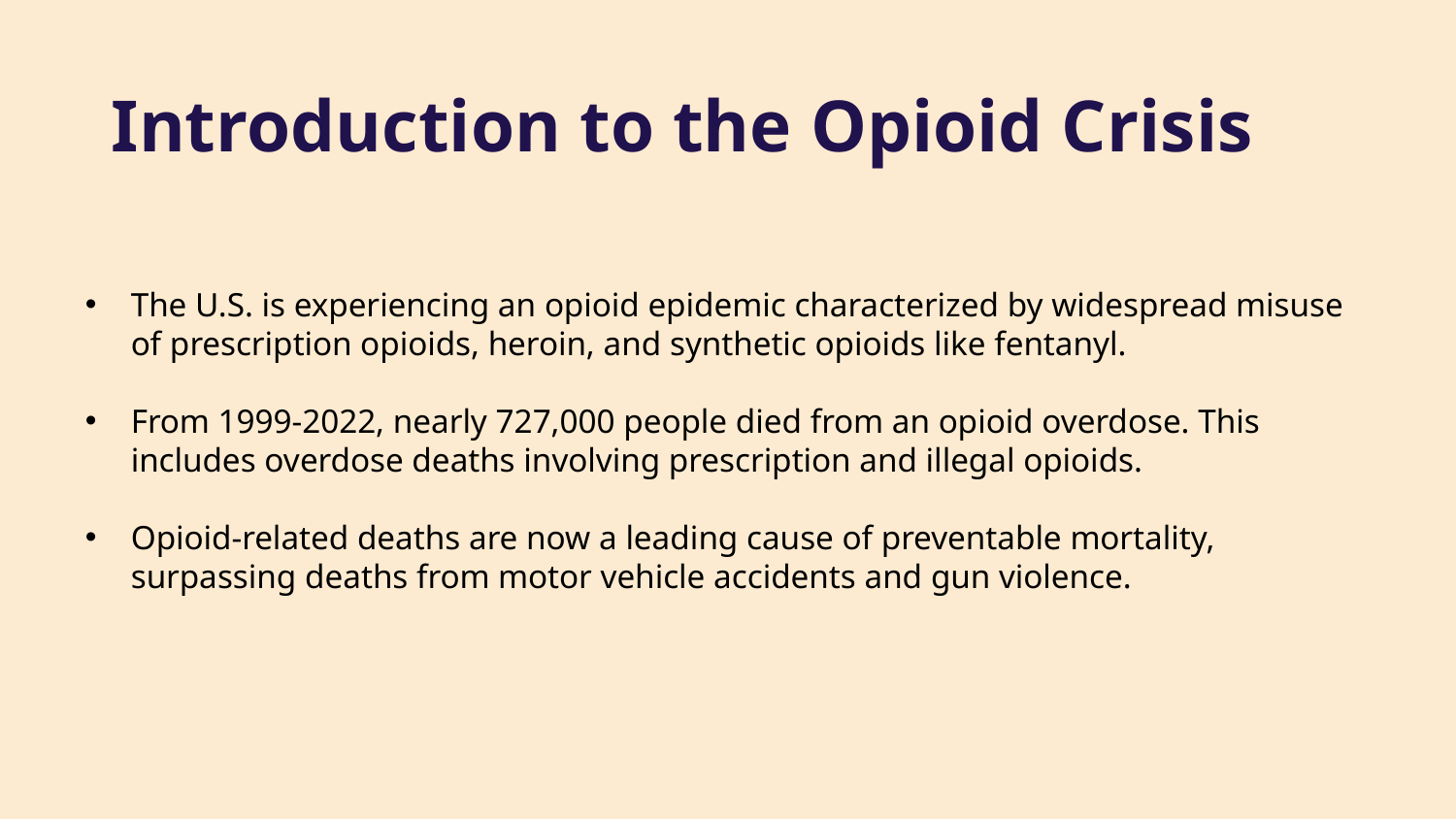

Introduction to the Opioid Crisis
The U.S. is experiencing an opioid epidemic characterized by widespread misuse of prescription opioids, heroin, and synthetic opioids like fentanyl.
From 1999-2022, nearly 727,000 people died from an opioid overdose. This includes overdose deaths involving prescription and illegal opioids.
Opioid-related deaths are now a leading cause of preventable mortality, surpassing deaths from motor vehicle accidents and gun violence.

## Slide 3
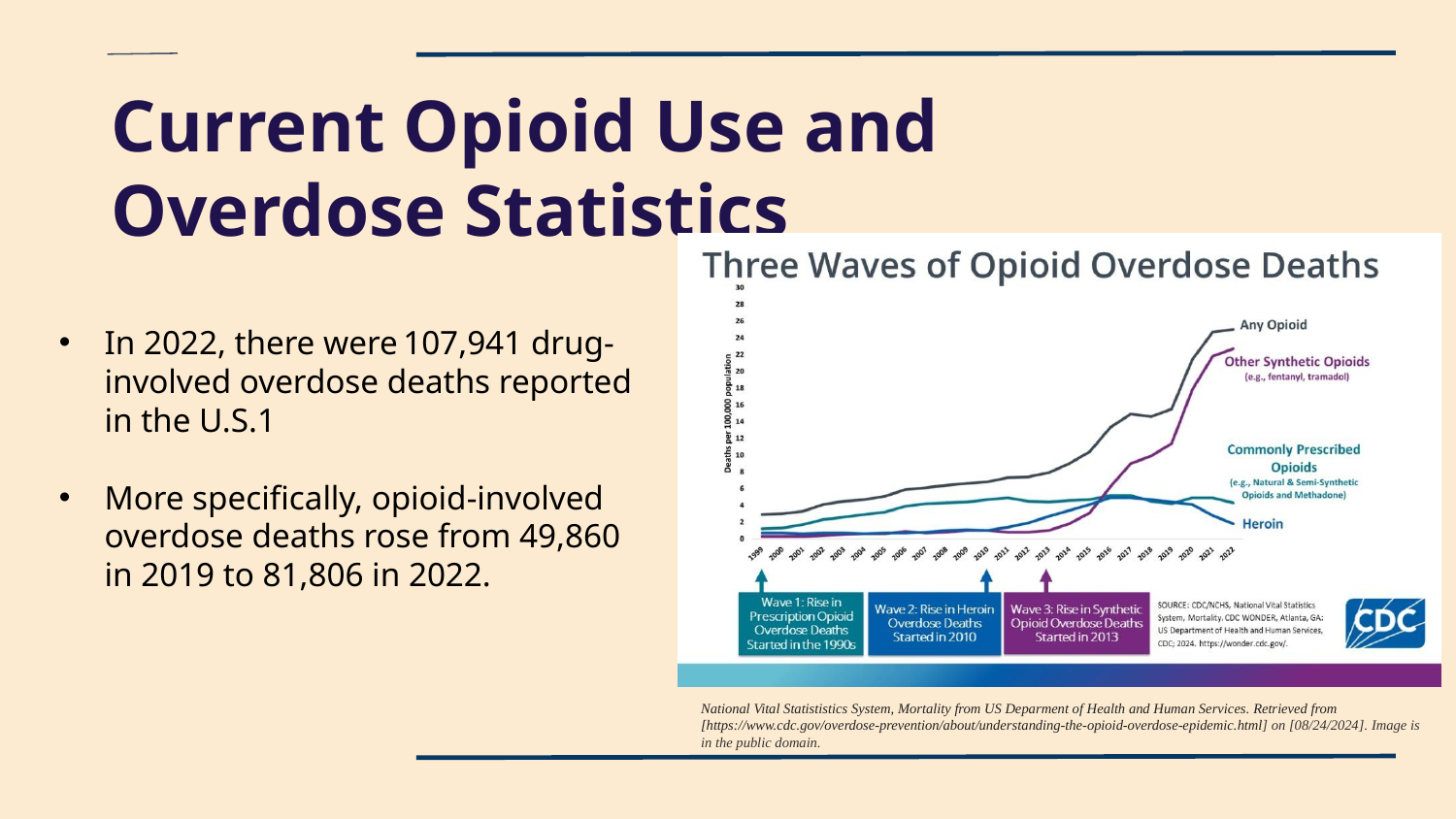

Current Opioid Use and Overdose Statistics
In 2022, there were 107,941 drug-involved overdose deaths reported in the U.S.1
More specifically, opioid-involved overdose deaths rose from 49,860 in 2019 to 81,806 in 2022.
Insert visuals: Infographic or data chart showing the increase in opioid-related deaths over the years.
National Vital Statististics System, Mortality from US Deparment of Health and Human Services. Retrieved from [https://www.cdc.gov/overdose-prevention/about/understanding-the-opioid-overdose-epidemic.html] on [08/24/2024]. Image is in the public domain.

## Slide 4
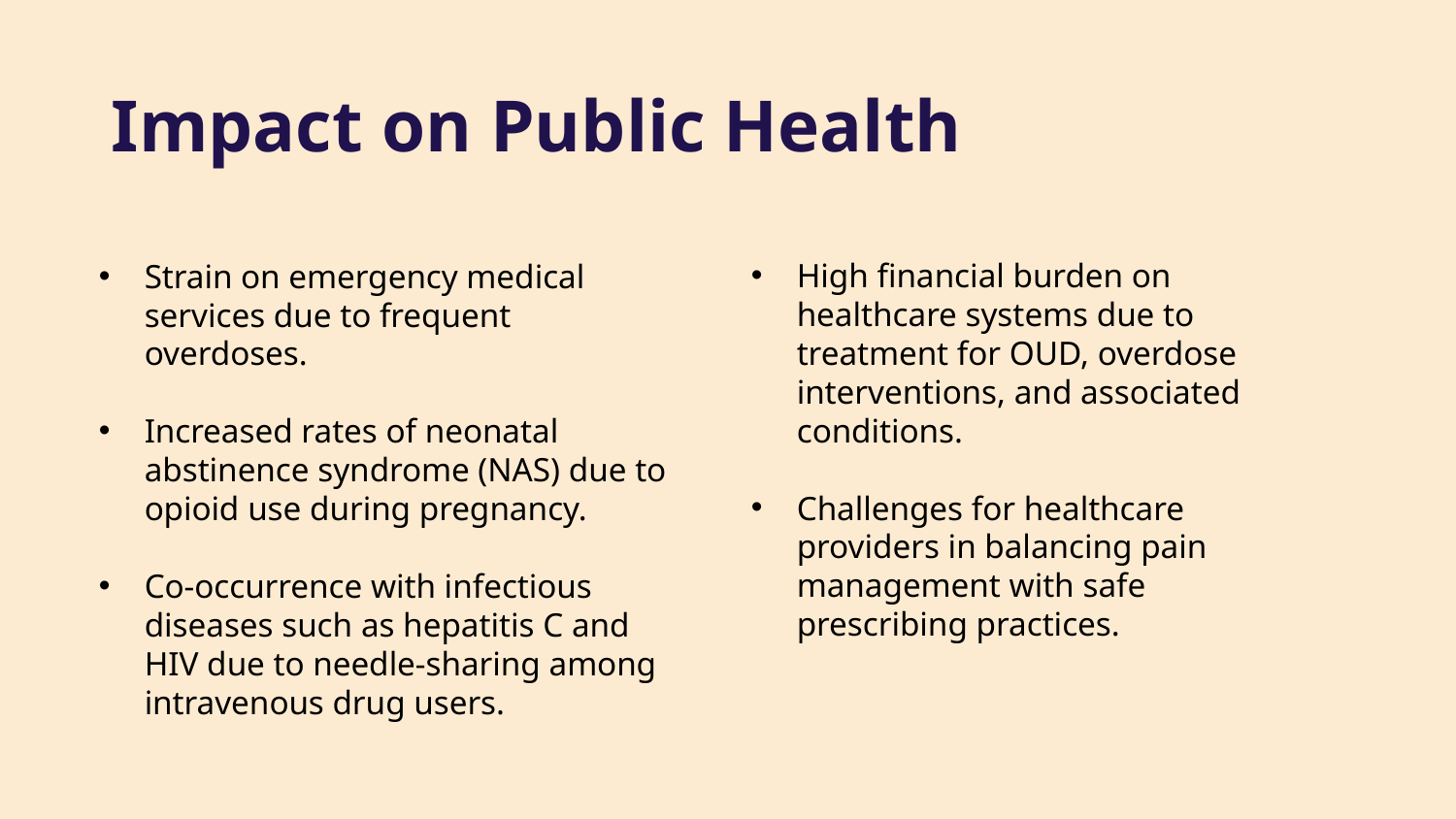

Impact on Public Health
High financial burden on healthcare systems due to treatment for OUD, overdose interventions, and associated conditions.
Challenges for healthcare providers in balancing pain management with safe prescribing practices.
Strain on emergency medical services due to frequent overdoses.
Increased rates of neonatal abstinence syndrome (NAS) due to opioid use during pregnancy.
Co-occurrence with infectious diseases such as hepatitis C and HIV due to needle-sharing among intravenous drug users.

## Slide 5
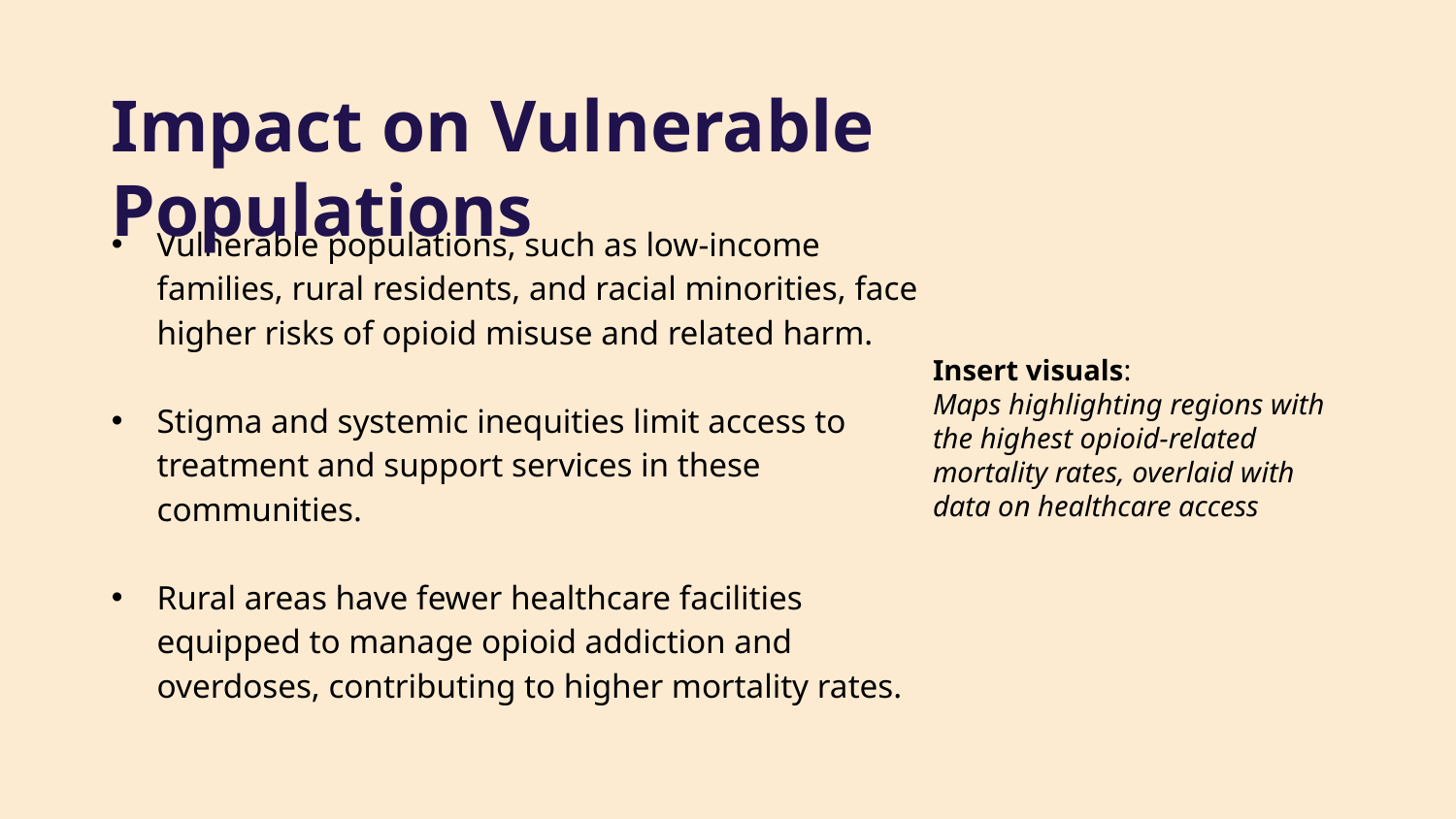

Impact on Vulnerable Populations
Vulnerable populations, such as low-income families, rural residents, and racial minorities, face higher risks of opioid misuse and related harm.
Stigma and systemic inequities limit access to treatment and support services in these communities.
Rural areas have fewer healthcare facilities equipped to manage opioid addiction and overdoses, contributing to higher mortality rates.
Insert visuals:
Maps highlighting regions with the highest opioid-related mortality rates, overlaid with data on healthcare access

## Slide 6
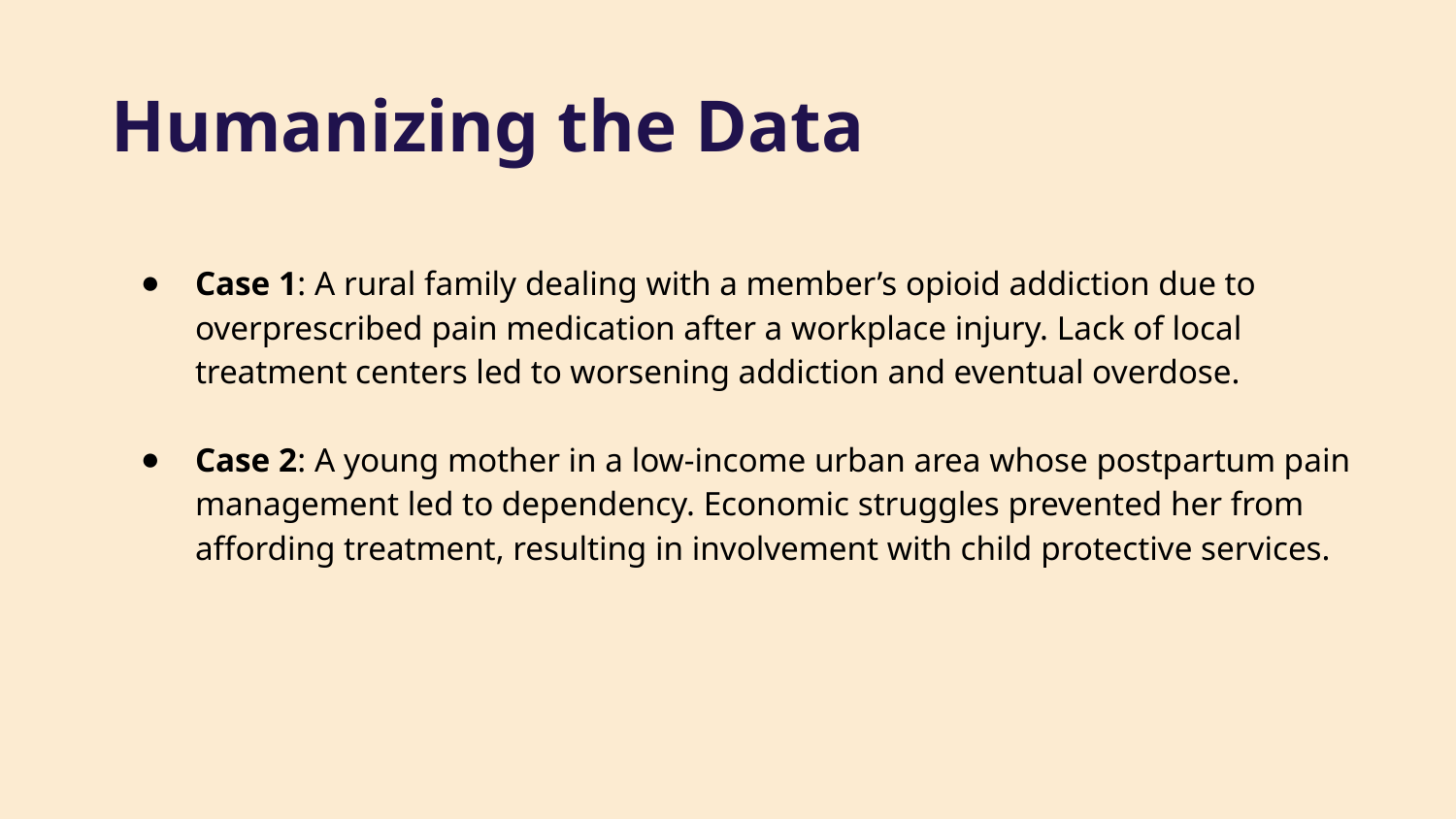

Humanizing the Data
Case 1: A rural family dealing with a member’s opioid addiction due to overprescribed pain medication after a workplace injury. Lack of local treatment centers led to worsening addiction and eventual overdose.
Case 2: A young mother in a low-income urban area whose postpartum pain management led to dependency. Economic struggles prevented her from affording treatment, resulting in involvement with child protective services.

## Slide 7
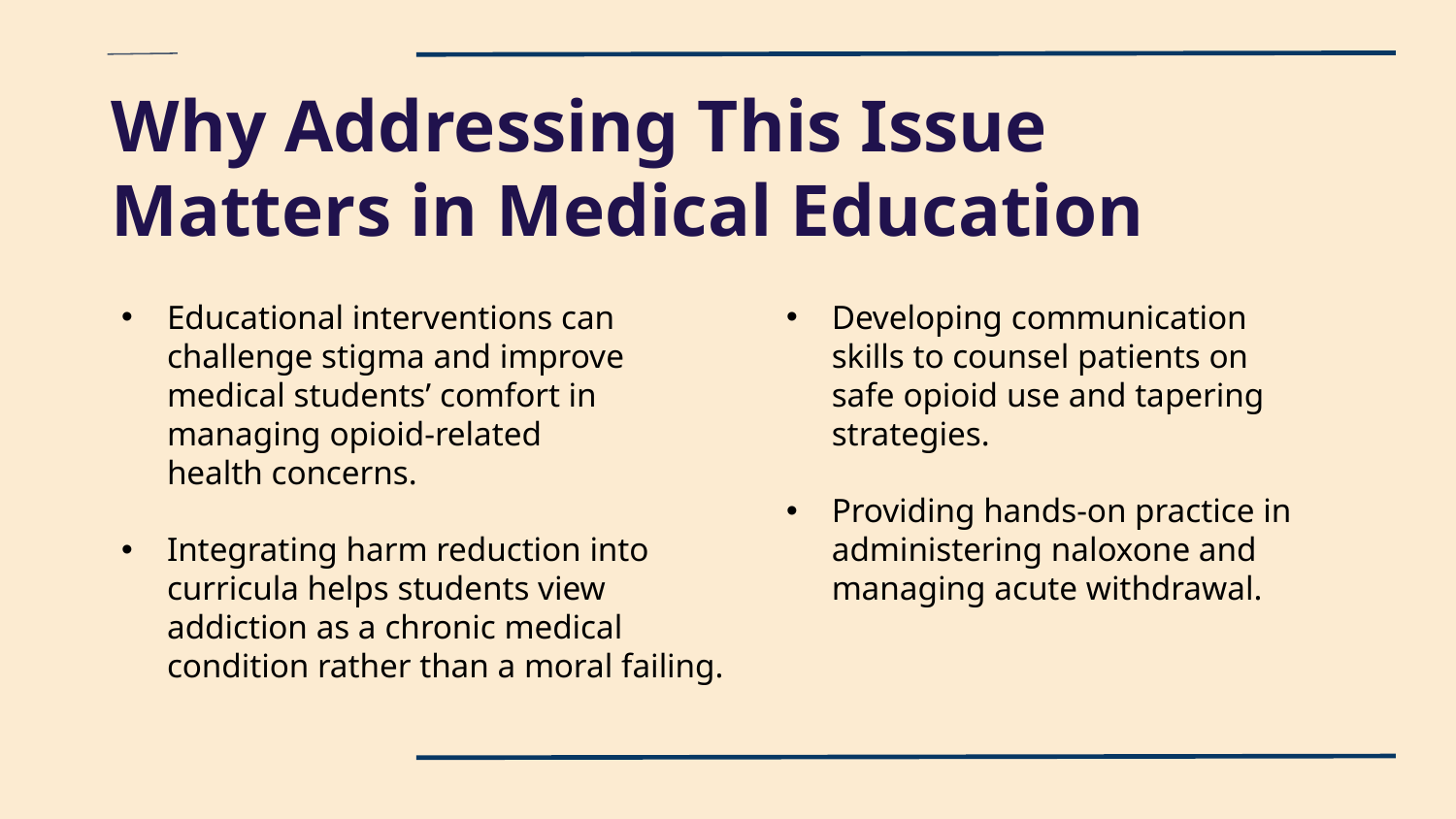

Why Addressing This Issue Matters in Medical Education
Educational interventions can challenge stigma and improve medical students’ comfort in managing opioid-related health concerns.
Integrating harm reduction into curricula helps students view addiction as a chronic medical condition rather than a moral failing.
Developing communication skills to counsel patients on safe opioid use and tapering strategies.
Providing hands-on practice in administering naloxone and managing acute withdrawal.

## Slide 8
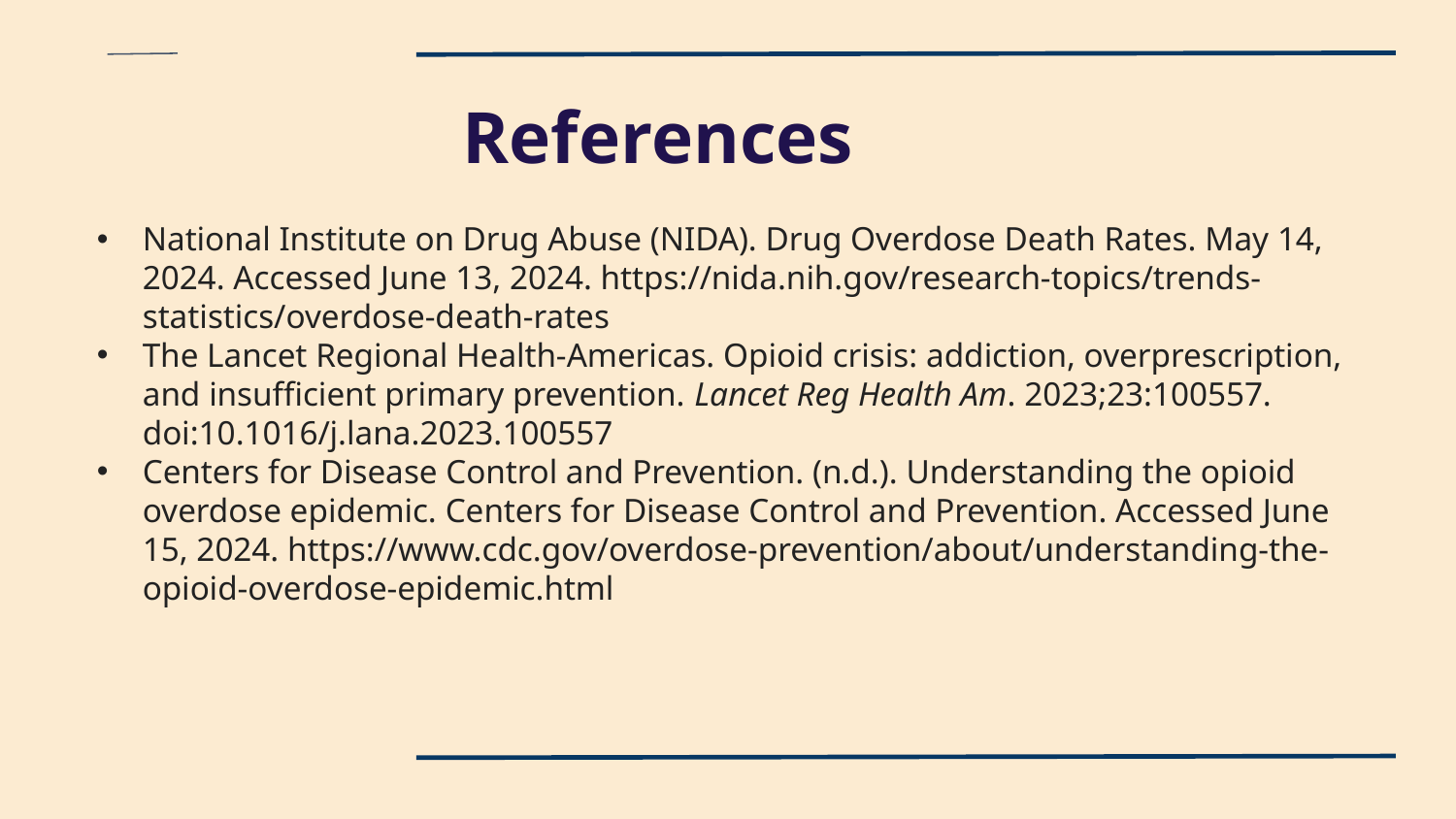

References
National Institute on Drug Abuse (NIDA). Drug Overdose Death Rates. May 14, 2024. Accessed June 13, 2024. https://nida.nih.gov/research-topics/trends-statistics/overdose-death-rates
The Lancet Regional Health-Americas. Opioid crisis: addiction, overprescription, and insufficient primary prevention. Lancet Reg Health Am. 2023;23:100557. doi:10.1016/j.lana.2023.100557
Centers for Disease Control and Prevention. (n.d.). Understanding the opioid overdose epidemic. Centers for Disease Control and Prevention. Accessed June 15, 2024. https://www.cdc.gov/overdose-prevention/about/understanding-the-opioid-overdose-epidemic.html

## Slide 9
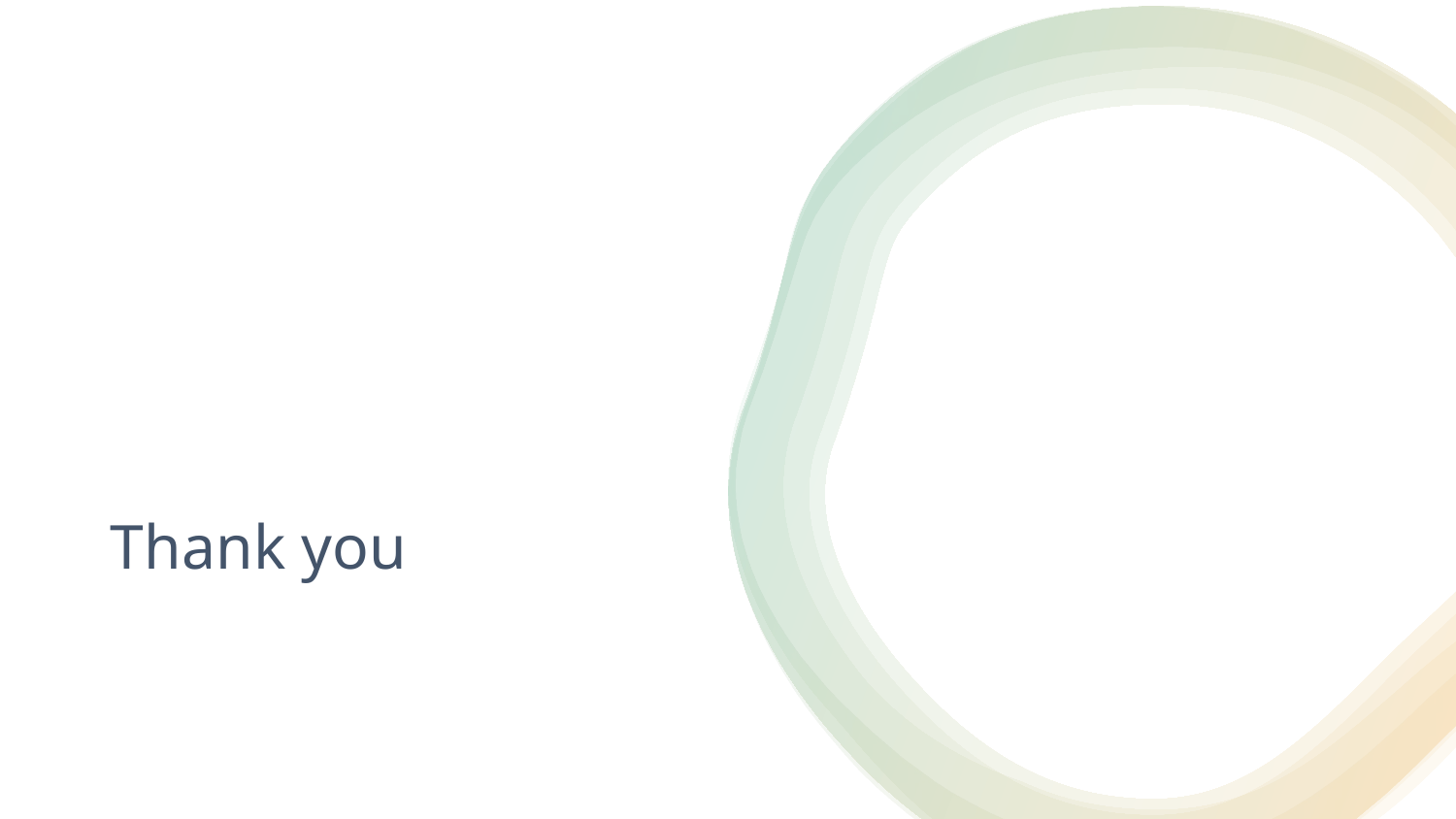

# Thank you
