# Supplementary material for: Empowering Future Physicians: Enhancing Naloxone Competency Through Early Harm Reduction Training in Medical Education
Source: MedEdPORTAL. 2025 Feb 14;21:11499. doi: 10.15766/mep_2374-8265.11499 (PMC11825861; doi:10.15766/mep_2374-8265.11499)
Supplement: Supplementary file 1 — Facilitator Guide.docxOpioid Overdose Statistics Lecture.pptxHarm Reduction Initiatives Lecture.pptxCase-Based Discussion Scenario.pptxOSCE-Style Checklist.docxTraining Session Confidence Survey.docx [file mep_2374-8265.11499-s001.zip › C. Harm Reduction Initiatives Lecture.pptx]

## Slide 1
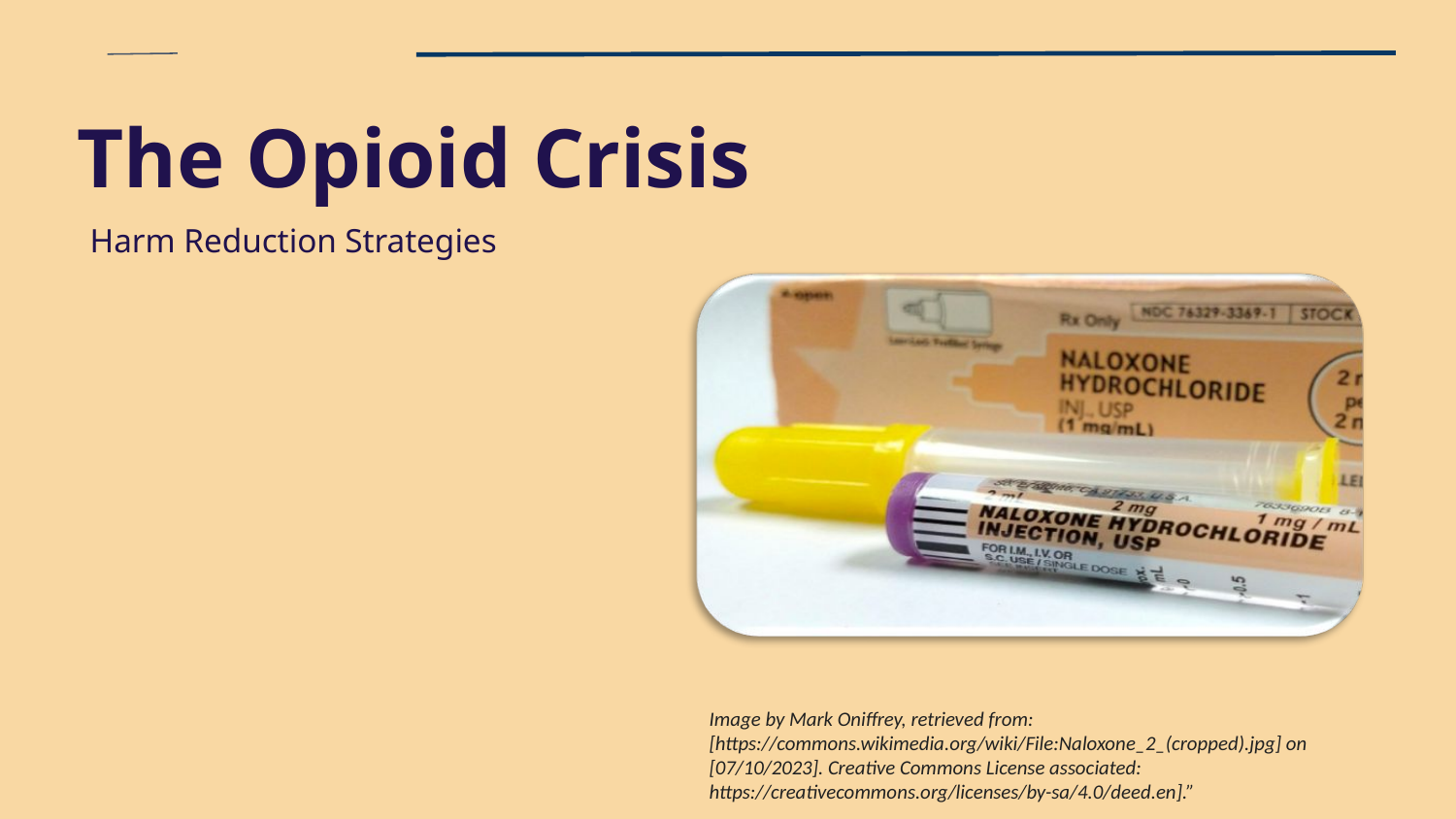

The Opioid Crisis
Harm Reduction Strategies​
Image by Mark Oniffrey, retrieved from: [https://commons.wikimedia.org/wiki/File:Naloxone_2_(cropped).jpg] on [07/10/2023]. Creative Commons License associated: https://creativecommons.org/licenses/by-sa/4.0/deed.en].”

## Slide 2
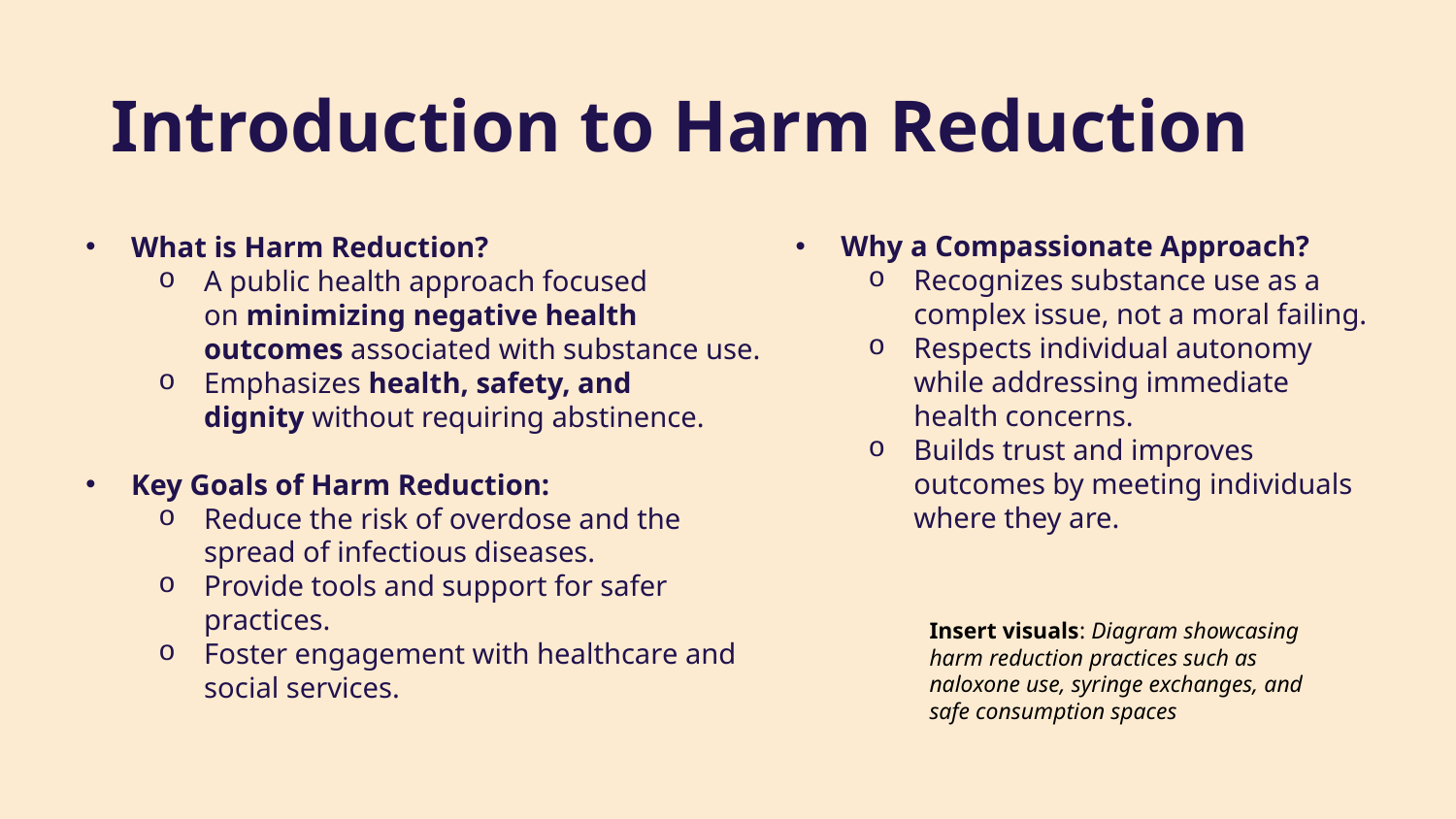

Introduction to Harm Reduction
Why a Compassionate Approach?​
Recognizes substance use as a complex issue, not a moral failing.​
Respects individual autonomy while addressing immediate health concerns.​
Builds trust and improves outcomes by meeting individuals where they are.
What is Harm Reduction?
A public health approach focused on minimizing negative health outcomes associated with substance use.
Emphasizes health, safety, and dignity without requiring abstinence.
Key Goals of Harm Reduction:
Reduce the risk of overdose and the spread of infectious diseases.
Provide tools and support for safer practices.
Foster engagement with healthcare and social services.
Insert visuals: Diagram showcasing harm reduction practices such as naloxone use, syringe exchanges, and safe consumption spaces

## Slide 3
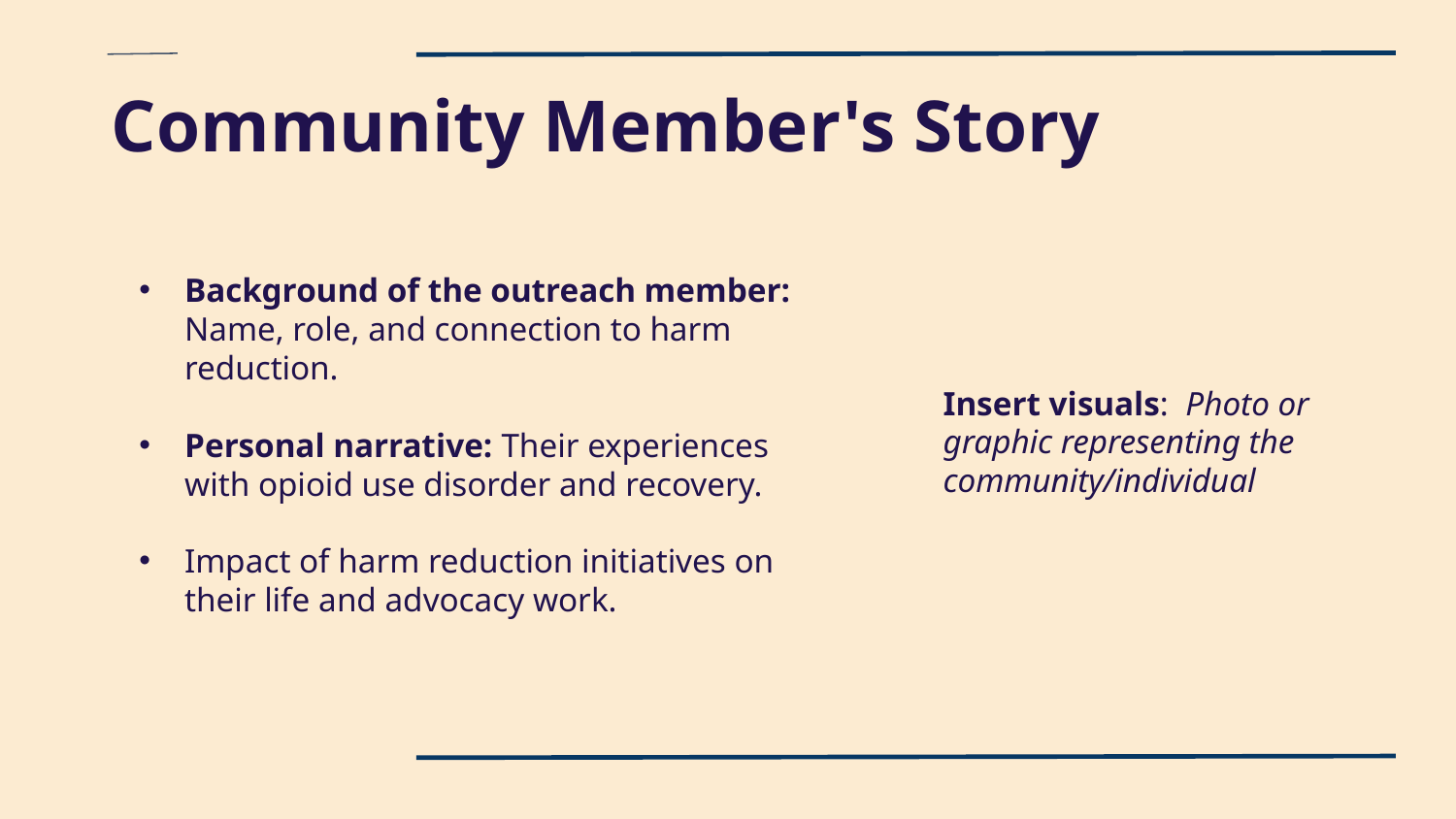

Community Member's Story
Background of the outreach member: Name, role, and connection to harm reduction.
Personal narrative: Their experiences with opioid use disorder and recovery.
Impact of harm reduction initiatives on their life and advocacy work.
Insert visuals:  Photo or graphic representing the community/individual

## Slide 4
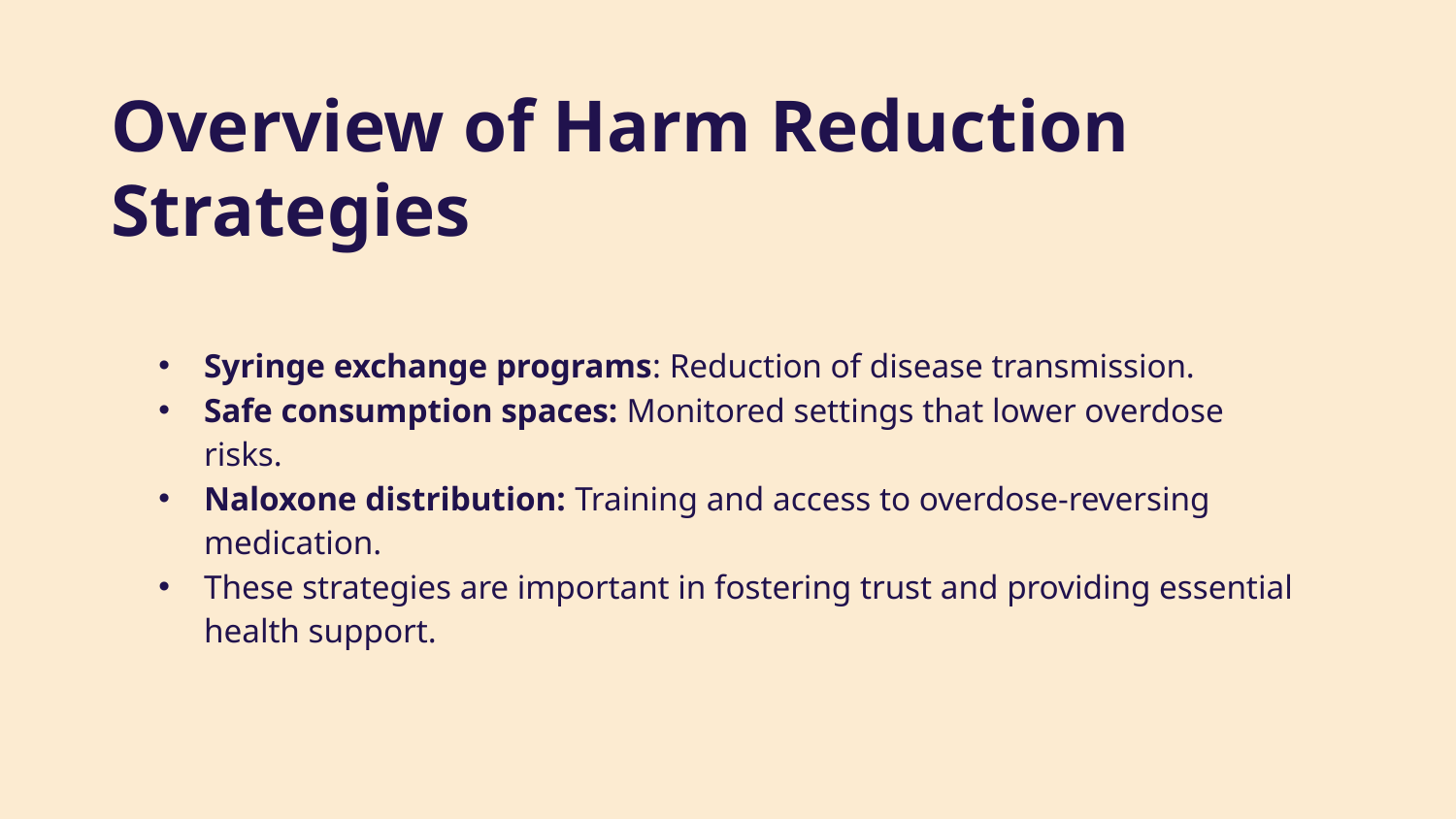

Overview of Harm Reduction Strategies
Syringe exchange programs: Reduction of disease transmission.
Safe consumption spaces: Monitored settings that lower overdose risks.
Naloxone distribution: Training and access to overdose-reversing medication.
These strategies are important in fostering trust and providing essential health support.

## Slide 5
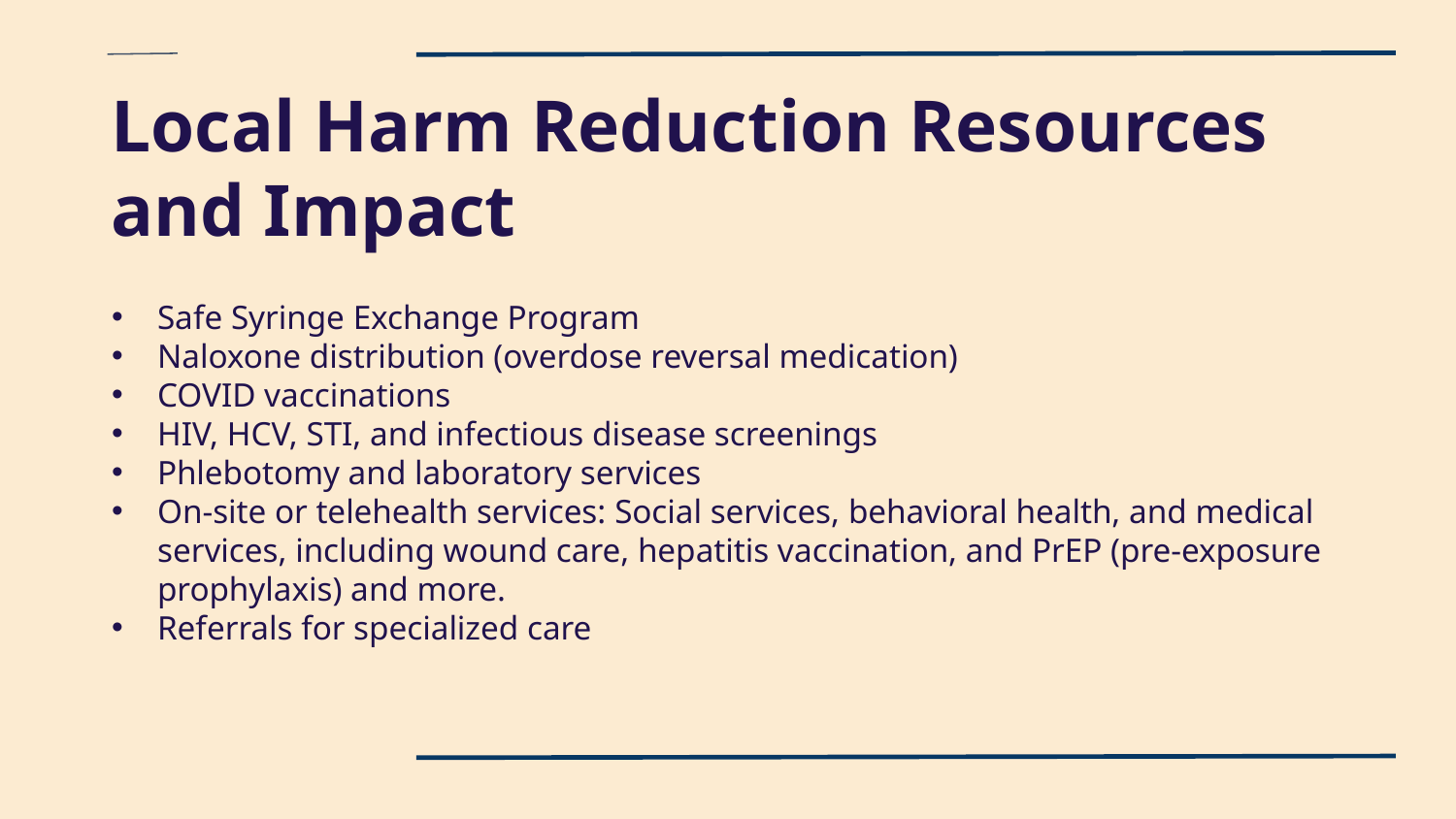

Local Harm Reduction Resources and Impact
Safe Syringe Exchange Program
Naloxone distribution (overdose reversal medication)
COVID vaccinations
HIV, HCV, STI, and infectious disease screenings
Phlebotomy and laboratory services
On-site or telehealth services: Social services, behavioral health, and medical services, including wound care, hepatitis vaccination, and PrEP (pre-exposure prophylaxis) and more.
Referrals for specialized care

## Slide 6
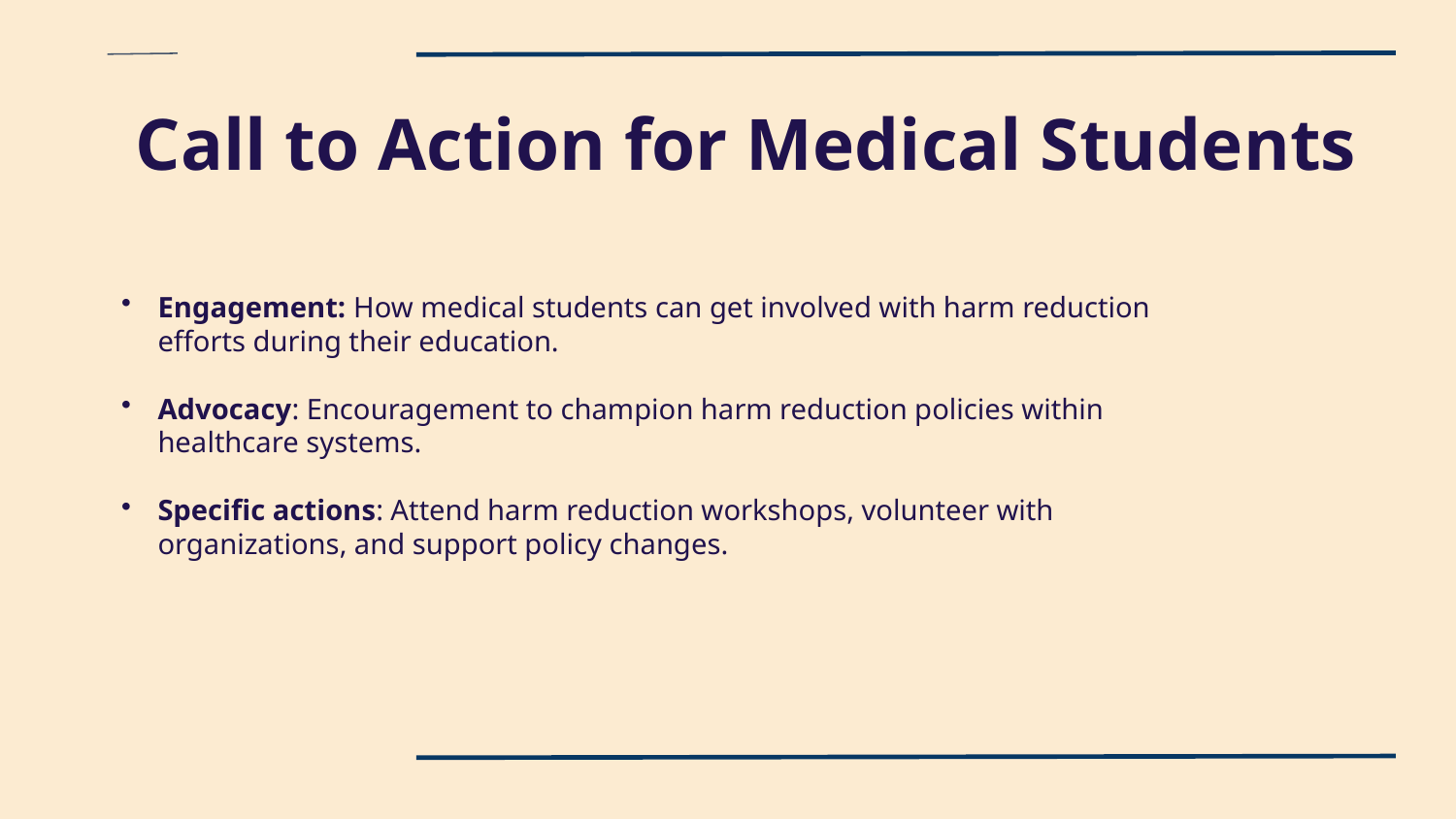

Call to Action for Medical Students
Engagement: How medical students can get involved with harm reduction efforts during their education.
Advocacy: Encouragement to champion harm reduction policies within healthcare systems.
Specific actions: Attend harm reduction workshops, volunteer with organizations, and support policy changes.

## Slide 7
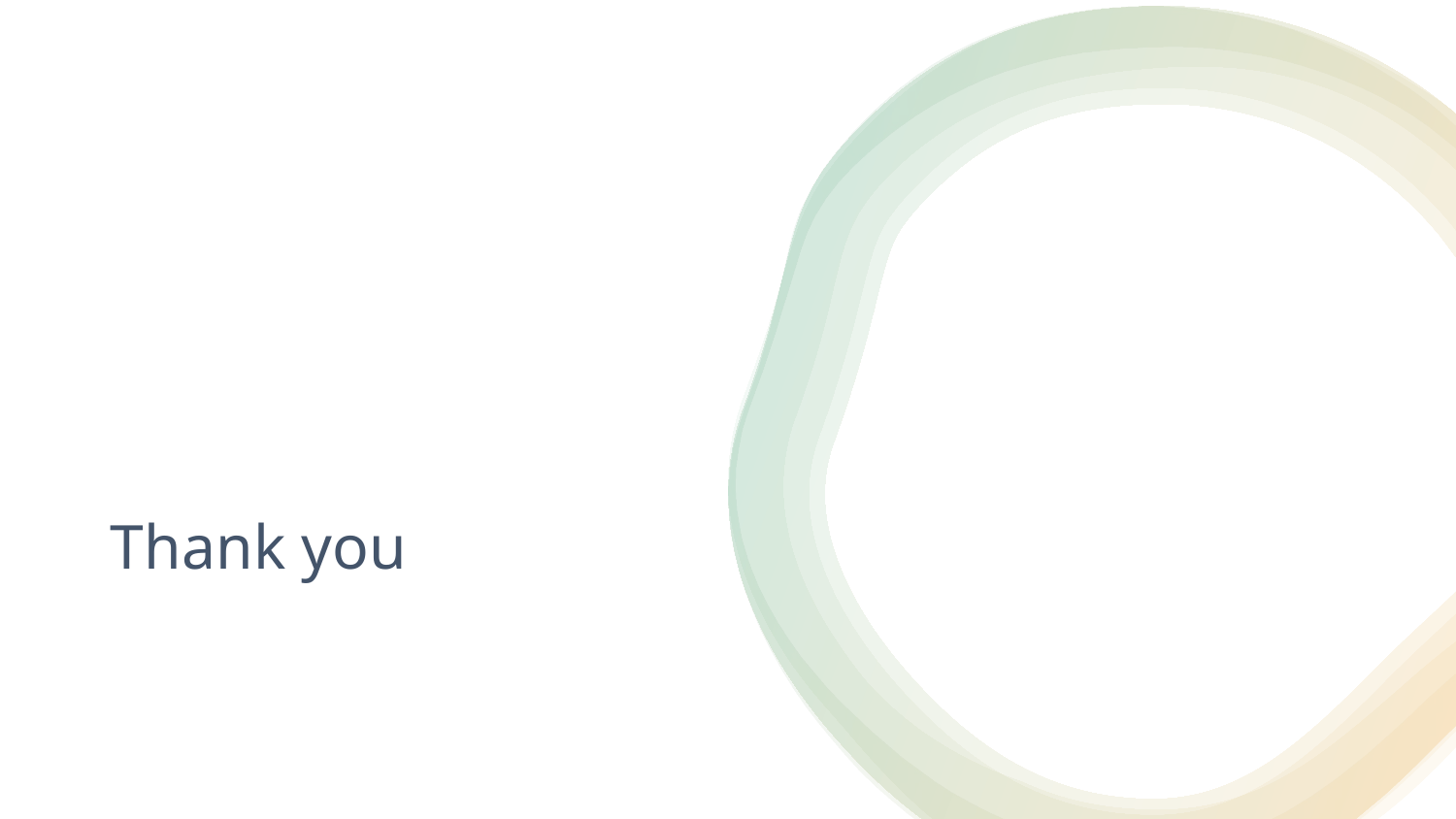

# Thank you
