# Supplementary material for: Empowering Future Physicians: Enhancing Naloxone Competency Through Early Harm Reduction Training in Medical Education
Source: MedEdPORTAL. 2025 Feb 14;21:11499. doi: 10.15766/mep_2374-8265.11499 (PMC11825861; doi:10.15766/mep_2374-8265.11499)
Supplement: Supplementary file 1 — Facilitator Guide.docxOpioid Overdose Statistics Lecture.pptxHarm Reduction Initiatives Lecture.pptxCase-Based Discussion Scenario.pptxOSCE-Style Checklist.docxTraining Session Confidence Survey.docx [file mep_2374-8265.11499-s001.zip › D. Case-Based Discussion Scenario.pptx]

## Slide 1
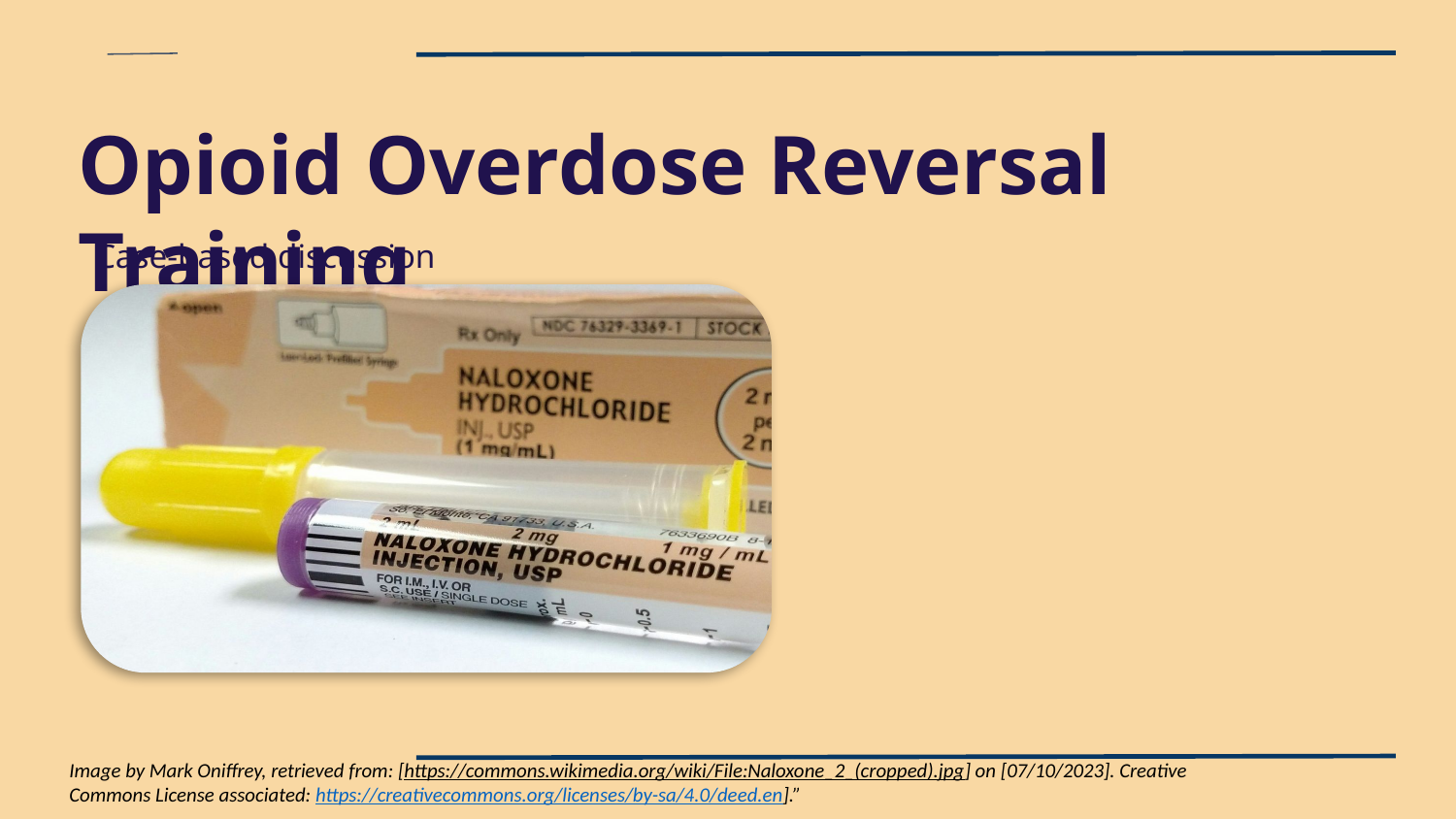

Opioid Overdose Reversal Training
Case-based discussion
Image by Mark Oniffrey, retrieved from: [https://commons.wikimedia.org/wiki/File:Naloxone_2_(cropped).jpg] on [07/10/2023]. Creative Commons License associated: https://creativecommons.org/licenses/by-sa/4.0/deed.en].”

## Slide 2
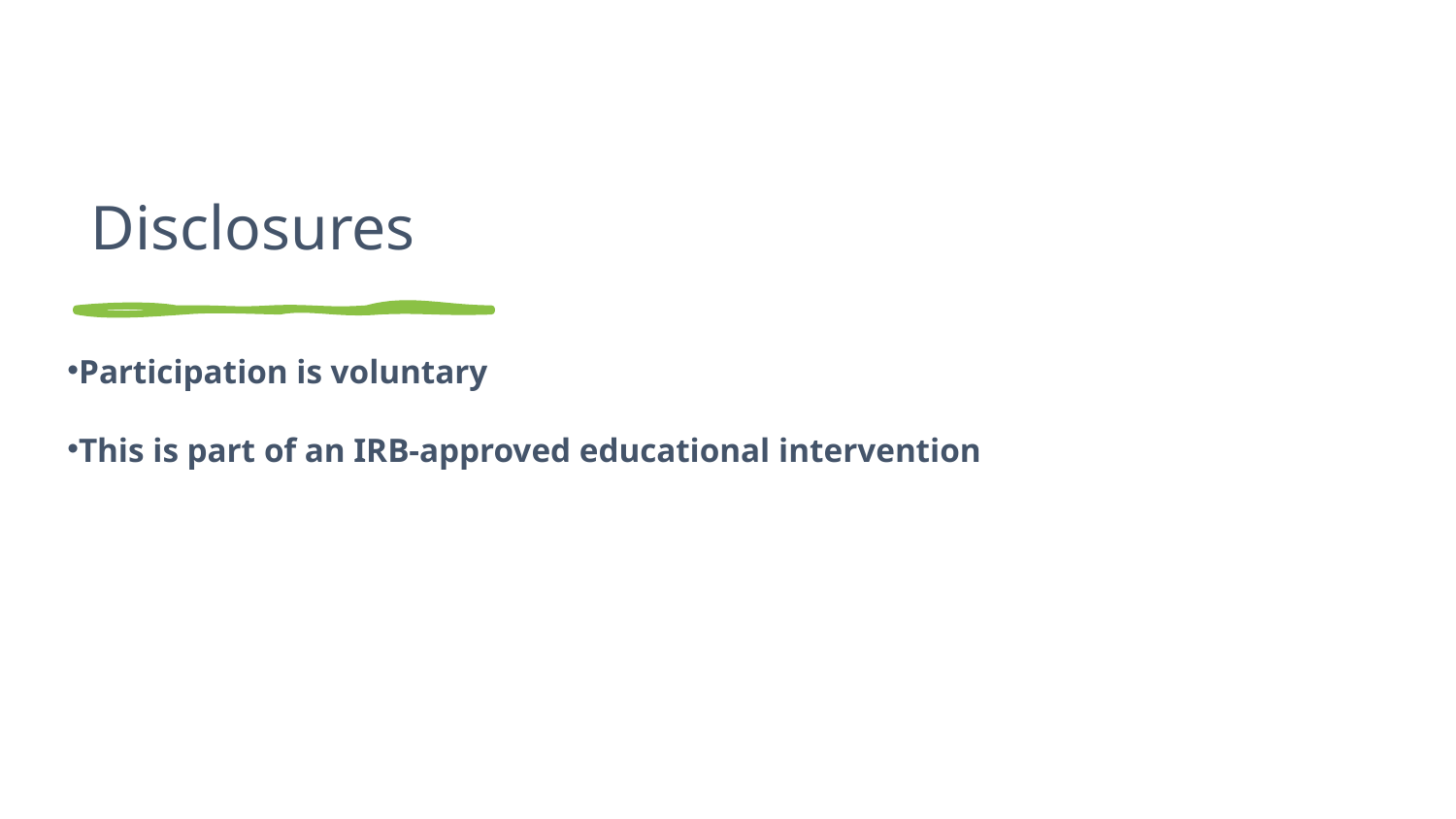

Disclosures
Participation is voluntary
This is part of an IRB-approved educational intervention

## Slide 3
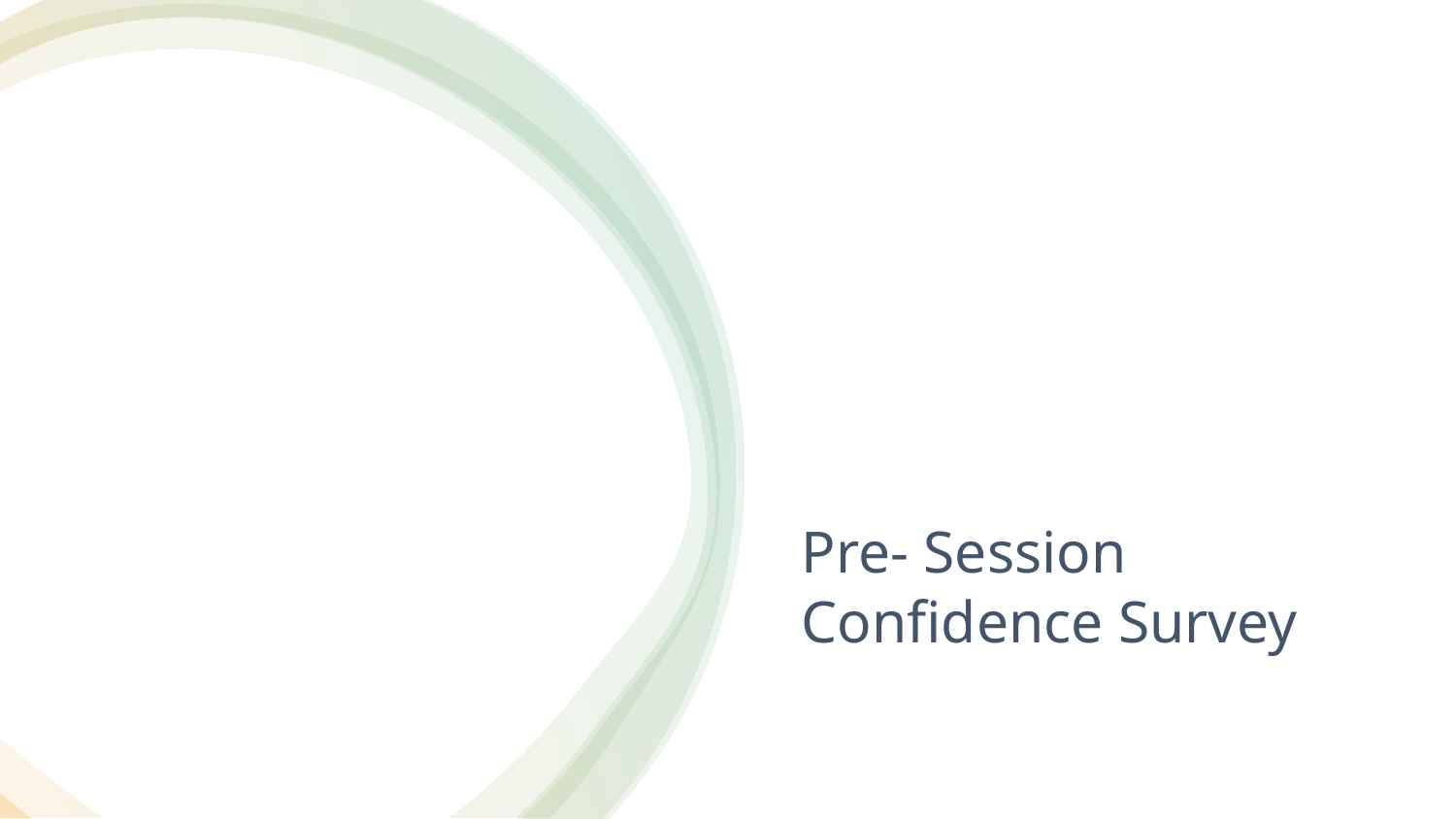

# Pre- Session Confidence Survey

## Slide 4
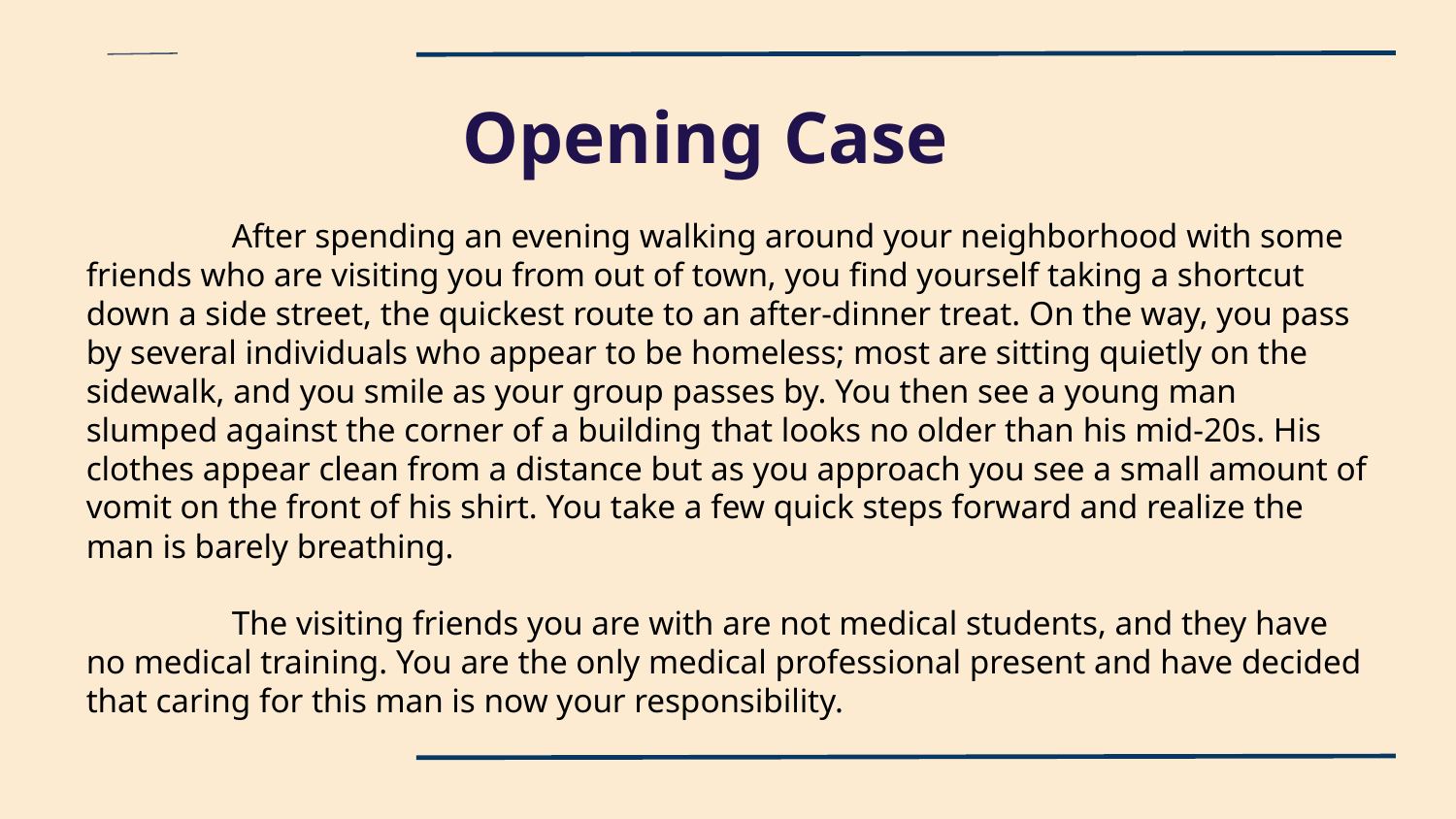

Opening Case
	After spending an evening walking around your neighborhood with some friends who are visiting you from out of town, you find yourself taking a shortcut down a side street, the quickest route to an after-dinner treat. On the way, you pass by several individuals who appear to be homeless; most are sitting quietly on the sidewalk, and you smile as your group passes by. You then see a young man slumped against the corner of a building that looks no older than his mid-20s. His clothes appear clean from a distance but as you approach you see a small amount of vomit on the front of his shirt. You take a few quick steps forward and realize the man is barely breathing.
	The visiting friends you are with are not medical students, and they have no medical training. You are the only medical professional present and have decided that caring for this man is now your responsibility.

## Slide 5
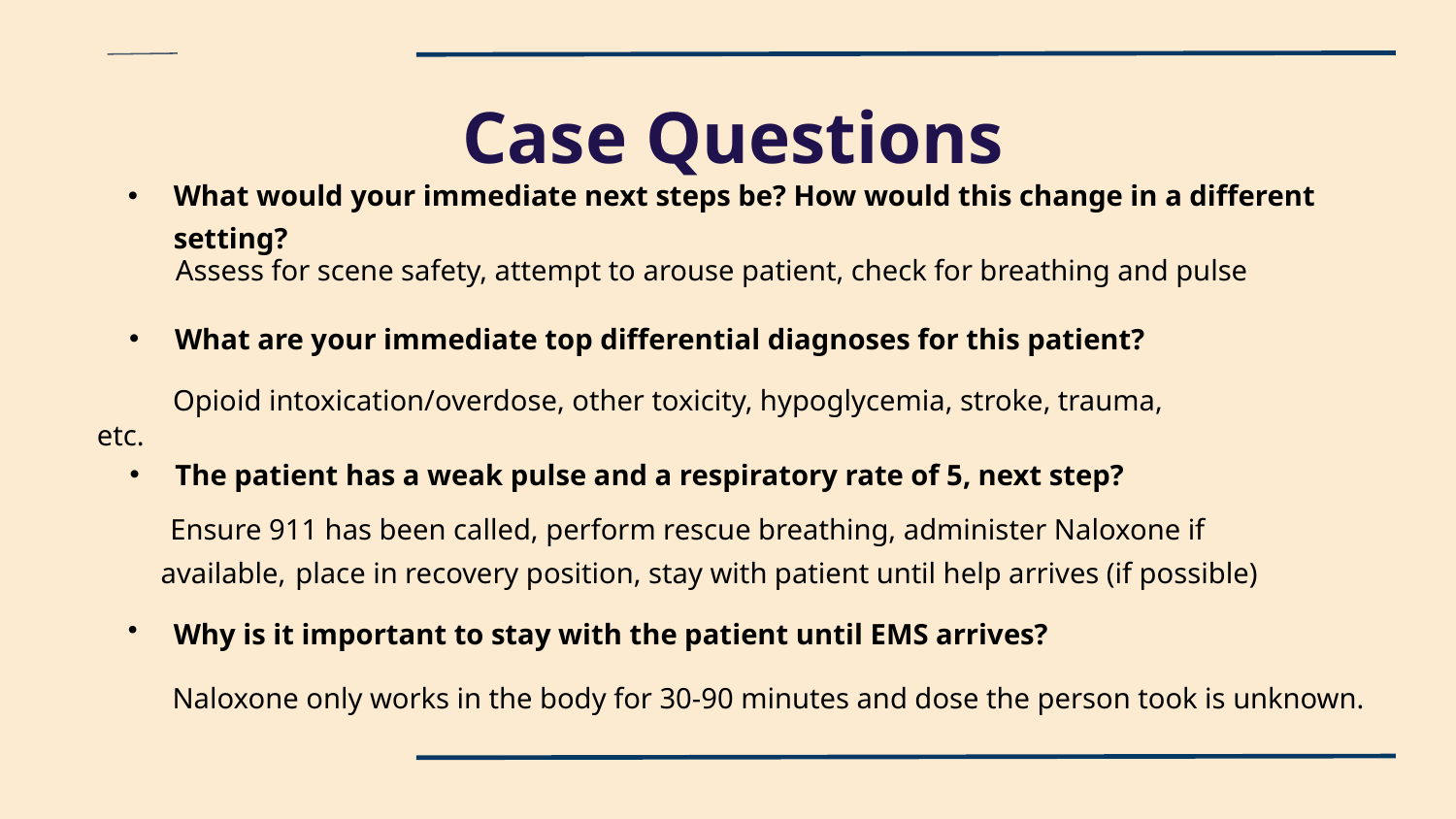

Case Questions
What would your immediate next steps be? How would this change in a different setting?
       Assess for scene safety, attempt to arouse patient, check for breathing and pulse
What are your immediate top differential diagnoses for this patient?
        Opioid intoxication/overdose, other toxicity, hypoglycemia, stroke, trauma, etc.
The patient has a weak pulse and a respiratory rate of 5, next step?
 Ensure 911 has been called, perform rescue breathing, administer Naloxone if available, place in recovery position, stay with patient until help arrives (if possible)
Why is it important to stay with the patient until EMS arrives?
     Naloxone only works in the body for 30-90 minutes and dose the person took is unknown.

## Slide 6
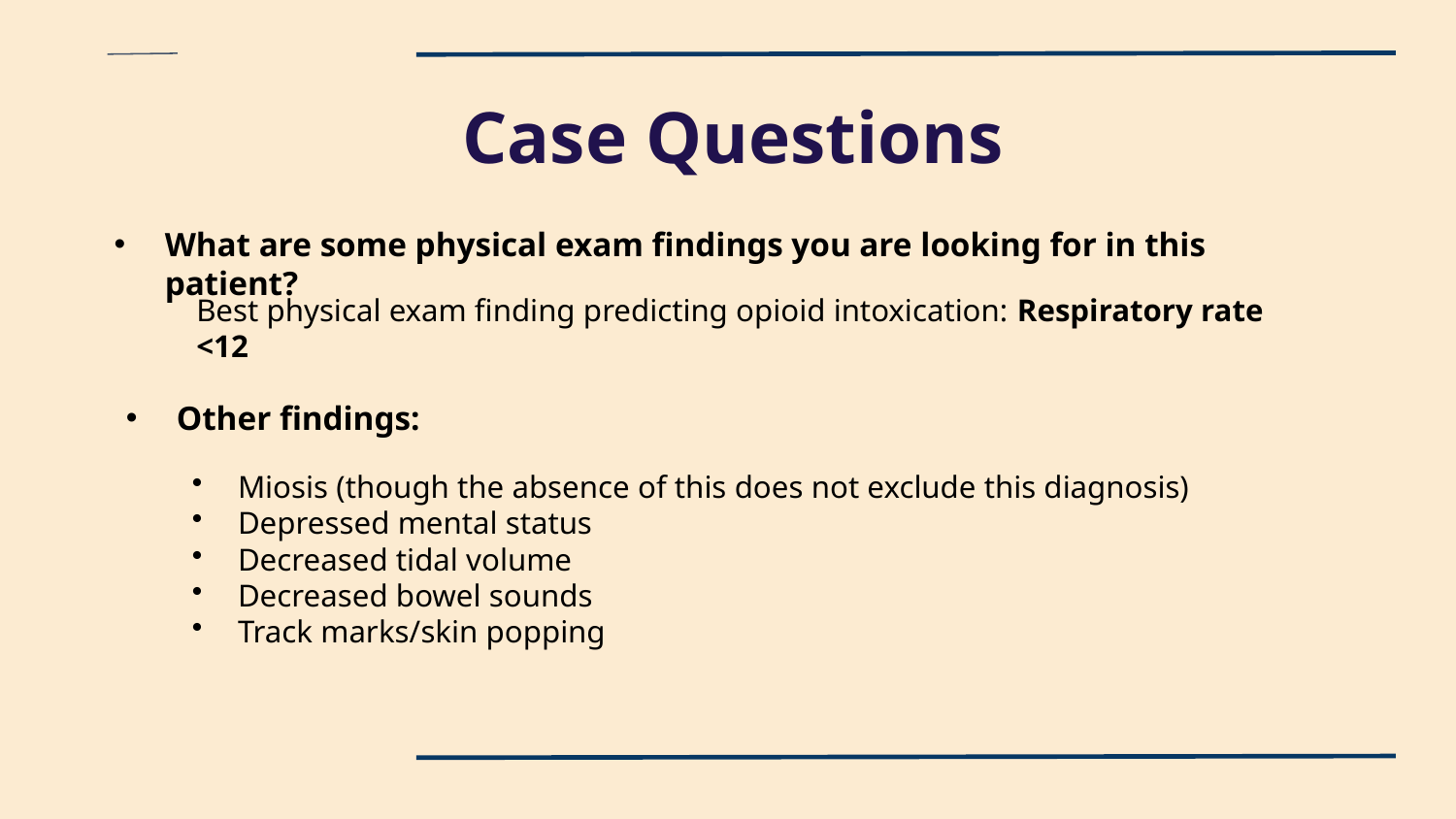

Case Questions
What are some physical exam findings you are looking for in this patient?
Best physical exam finding predicting opioid intoxication: Respiratory rate <12
Other findings:
Miosis (though the absence of this does not exclude this diagnosis)​​
Depressed mental status​​
Decreased tidal volume​​
Decreased bowel sounds​​
Track marks/skin popping

## Slide 7
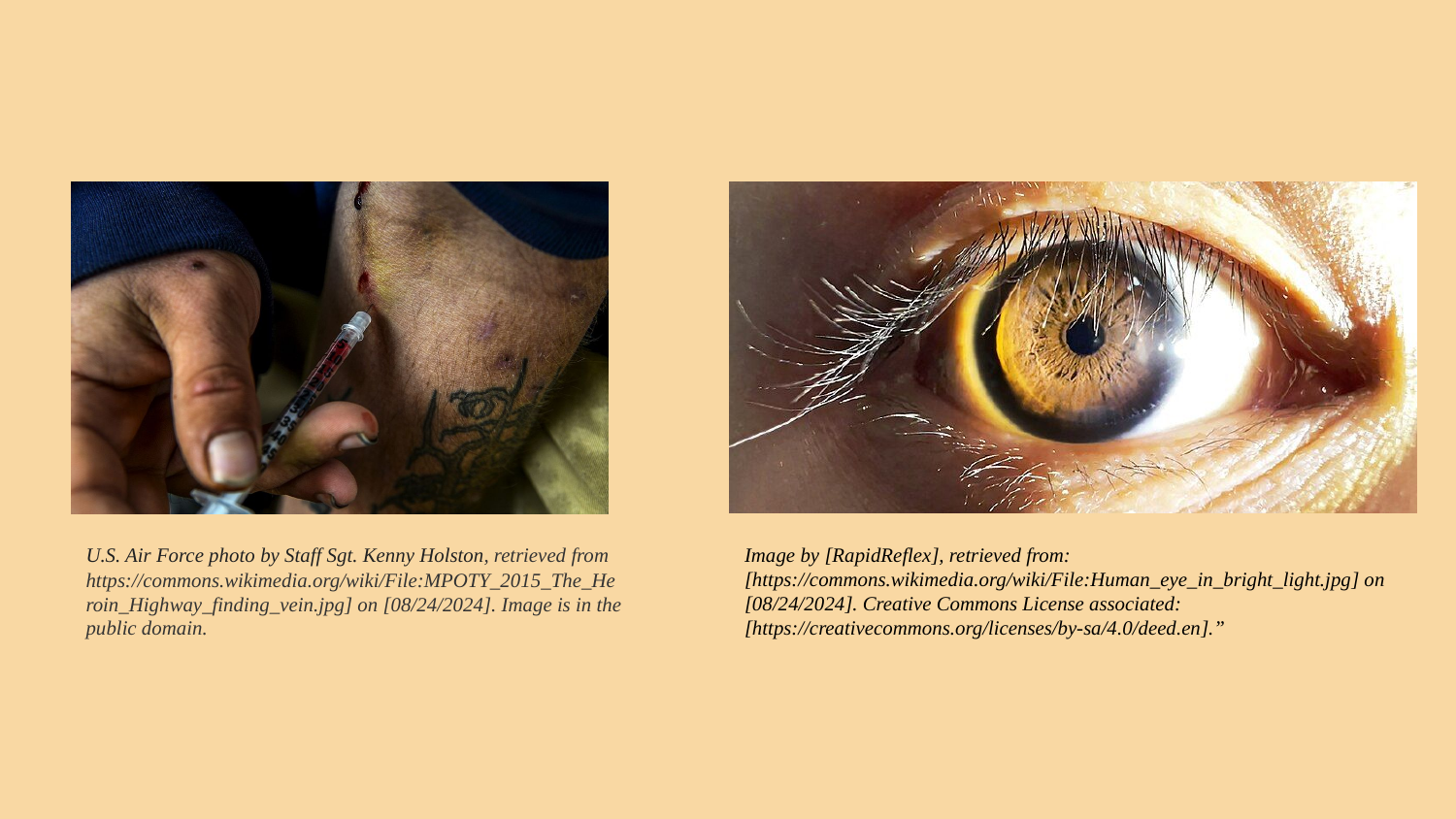

Image by [RapidReflex], retrieved from: [https://commons.wikimedia.org/wiki/File:Human_eye_in_bright_light.jpg] on [08/24/2024]. Creative Commons License associated: [https://creativecommons.org/licenses/by-sa/4.0/deed.en].”
U.S. Air Force photo by Staff Sgt. Kenny Holston, retrieved from https://commons.wikimedia.org/wiki/File:MPOTY_2015_The_Heroin_Highway_finding_vein.jpg] on [08/24/2024]. Image is in the public domain.

## Slide 8
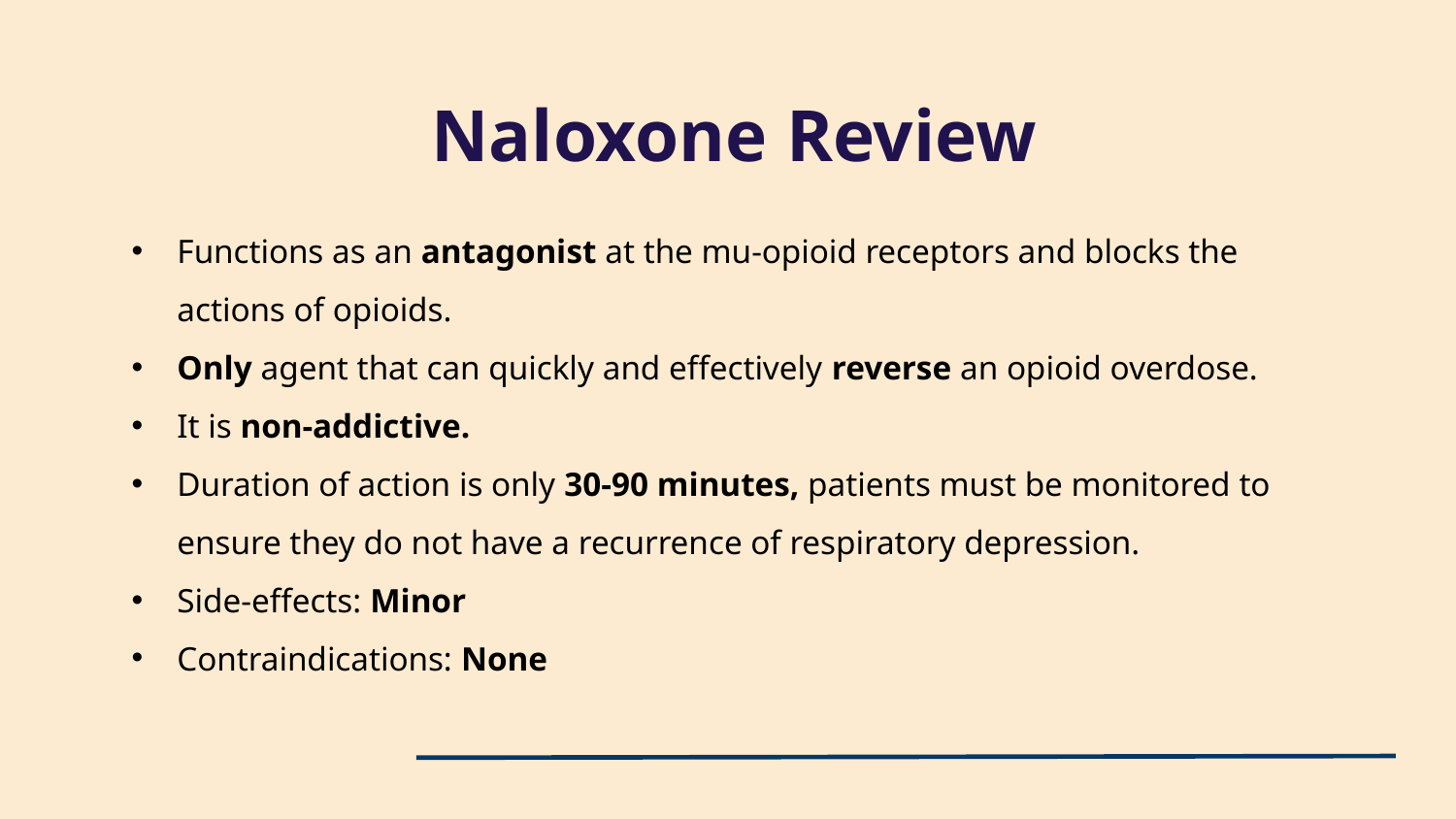

Naloxone Review
Functions as an antagonist at the mu-opioid receptors and blocks the actions of opioids.
Only agent that can quickly and effectively reverse an opioid overdose.
It is non-addictive.
Duration of action is only 30-90 minutes, patients must be monitored to ensure they do not have a recurrence of respiratory depression.
Side-effects: Minor
Contraindications: None

## Slide 9
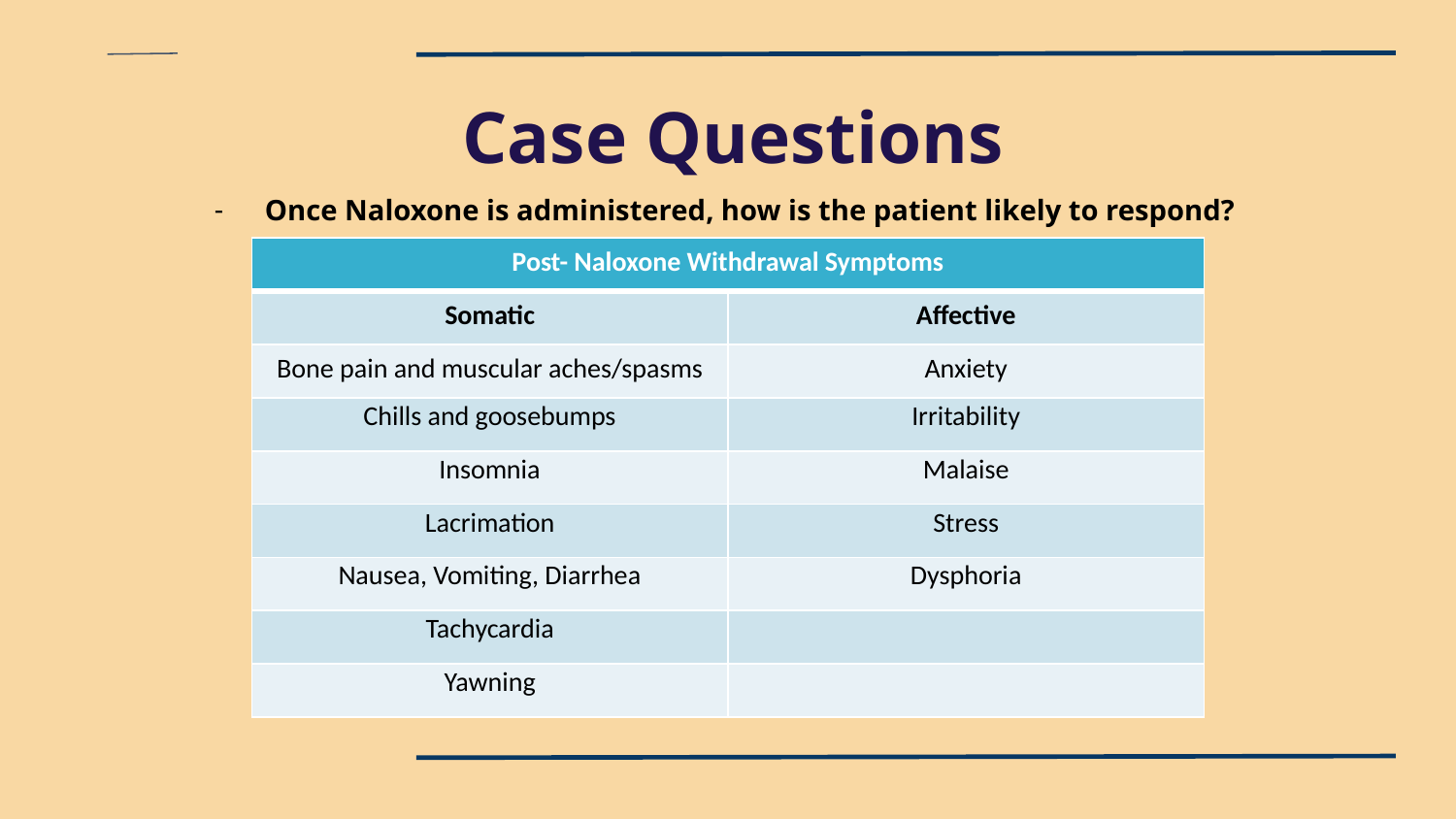

Case Questions
Once Naloxone is administered, how is the patient likely to respond?
| Post- Naloxone Withdrawal Symptoms | |
| --- | --- |
| Somatic | Affective |
| Bone pain and muscular aches/spasms | Anxiety |
| Chills and goosebumps | Irritability |
| Insomnia | Malaise |
| Lacrimation | Stress |
| Nausea, Vomiting, Diarrhea | Dysphoria |
| Tachycardia | |
| Yawning | |

## Slide 10
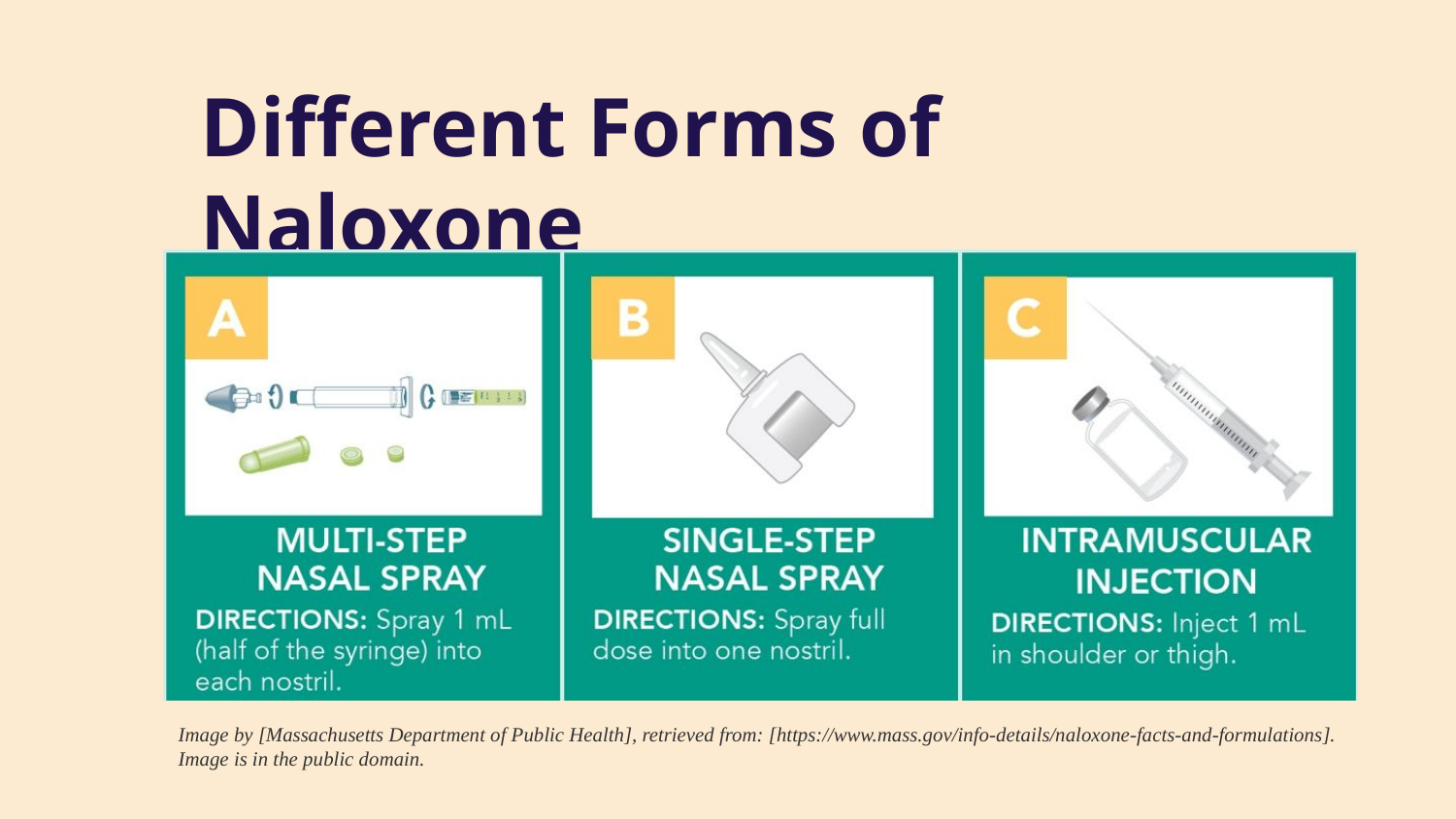

Different Forms of Naloxone
Image by [Massachusetts Department of Public Health], retrieved from: [https://www.mass.gov/info-details/naloxone-facts-and-formulations]. Image is in the public domain.

## Slide 11
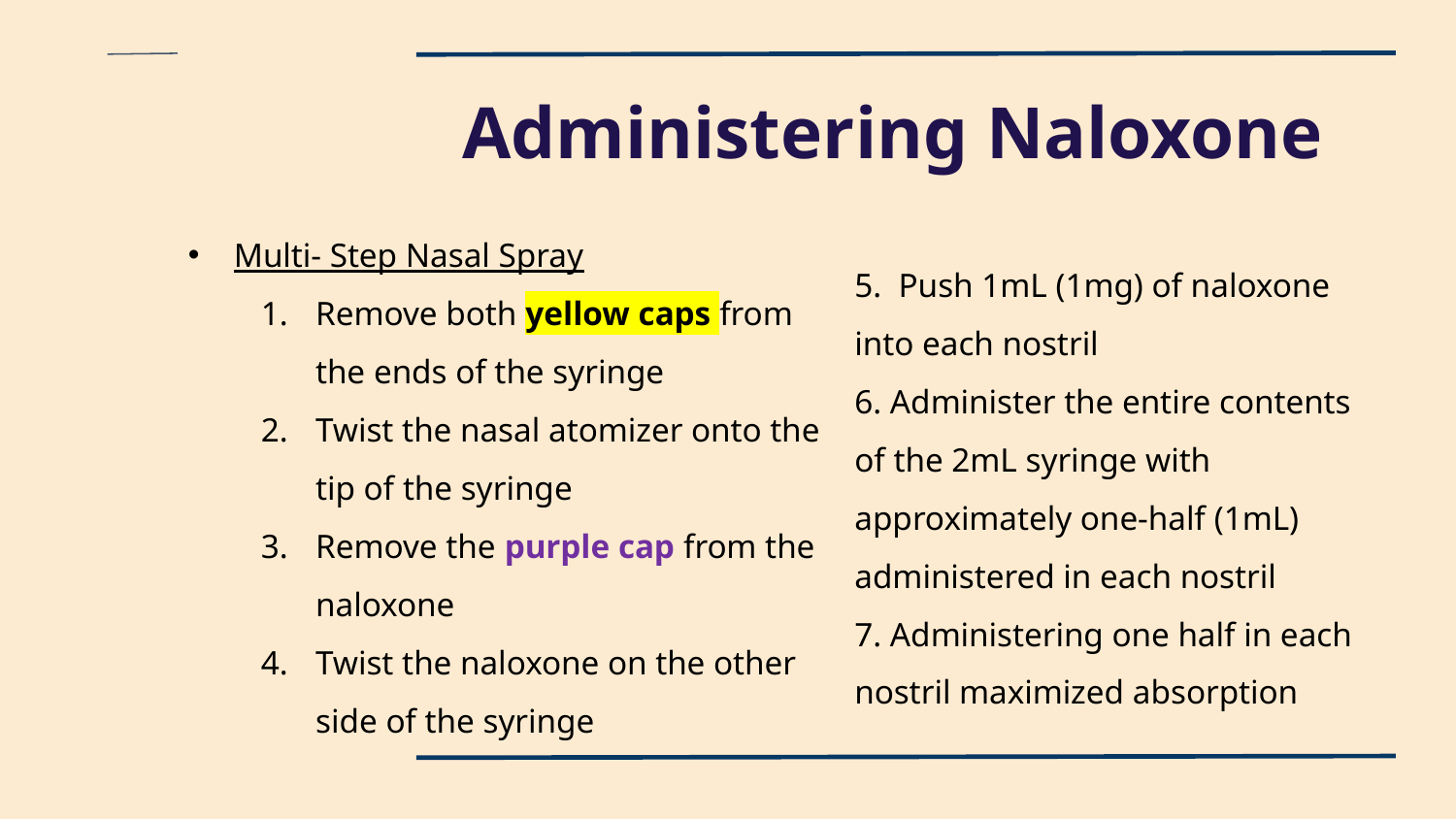

Administering Naloxone
5. Push 1mL (1mg) of naloxone into each nostril
6. Administer the entire contents of the 2mL syringe with approximately one-half (1mL) administered in each nostril
7. Administering one half in each nostril maximized absorption
Multi- Step Nasal Spray
Remove both yellow caps from the ends of the syringe
Twist the nasal atomizer onto the tip of the syringe
Remove the purple cap from the naloxone
Twist the naloxone on the other side of the syringe

## Slide 12
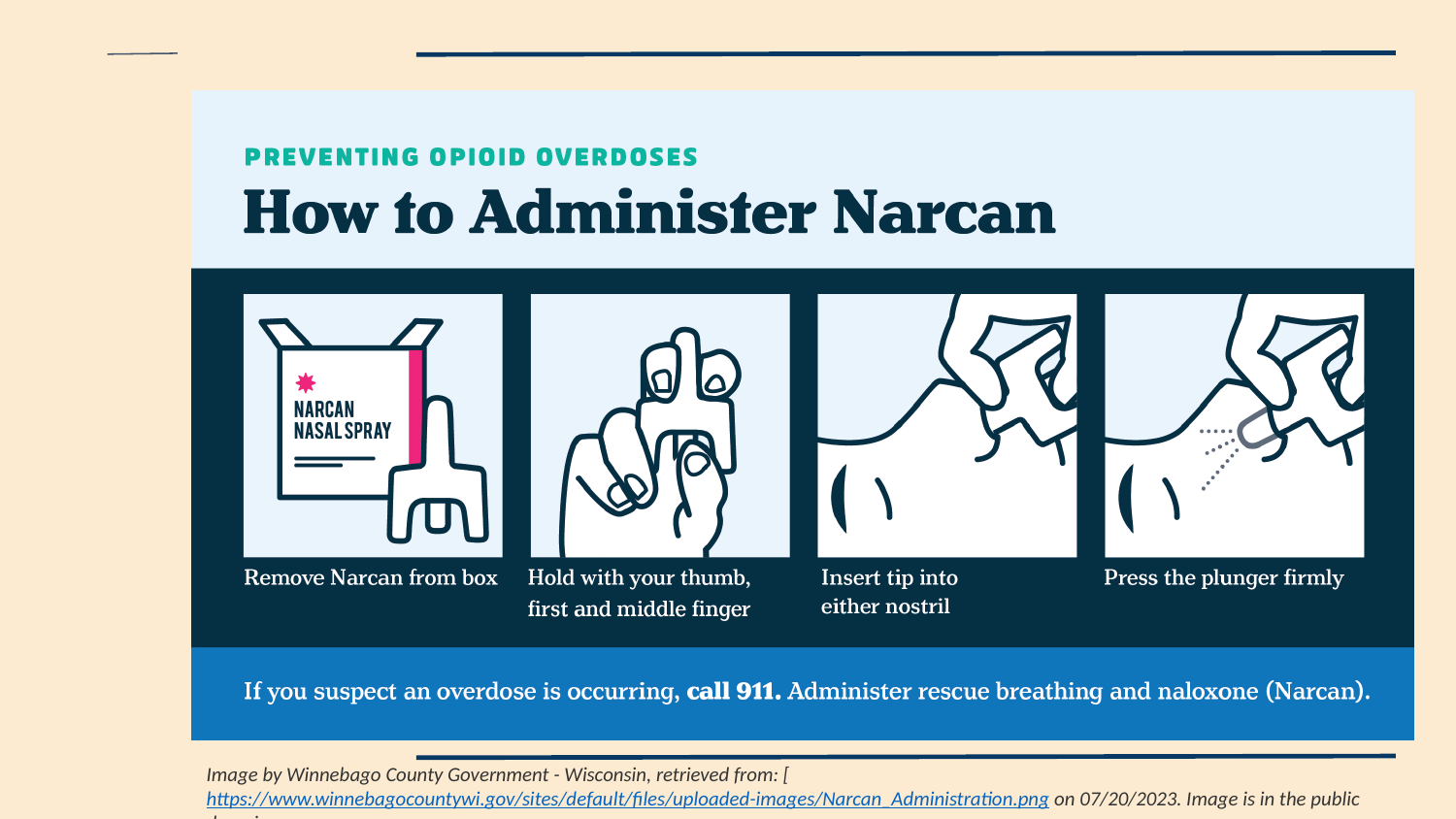

Administering Naloxone
Image by Winnebago County Government - Wisconsin, retrieved from: [https://www.winnebagocountywi.gov/sites/default/files/uploaded-images/Narcan_Administration.png on 07/20/2023. Image is in the public domain.

## Slide 13
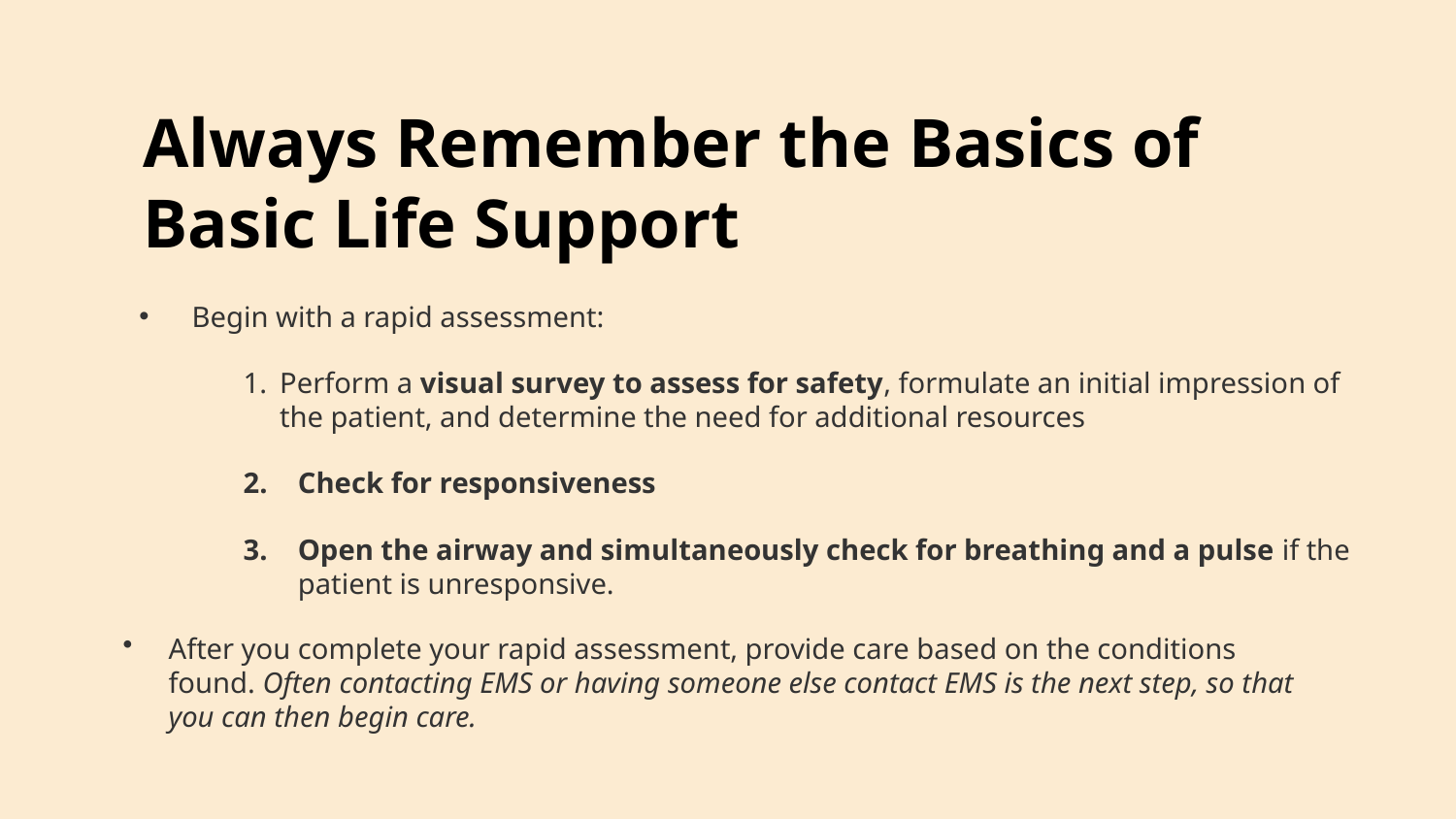

# Always Remember the Basics of Basic Life Support
 Begin with a rapid assessment:
Perform a visual survey to assess for safety, formulate an initial impression of the patient, and determine the need for additional resources
Check for responsiveness
Open the airway and simultaneously check for breathing and a pulse if the patient is unresponsive.
After you complete your rapid assessment, provide care based on the conditions found. Often contacting EMS or having someone else contact EMS is the next step, so that you can then begin care.

## Slide 14
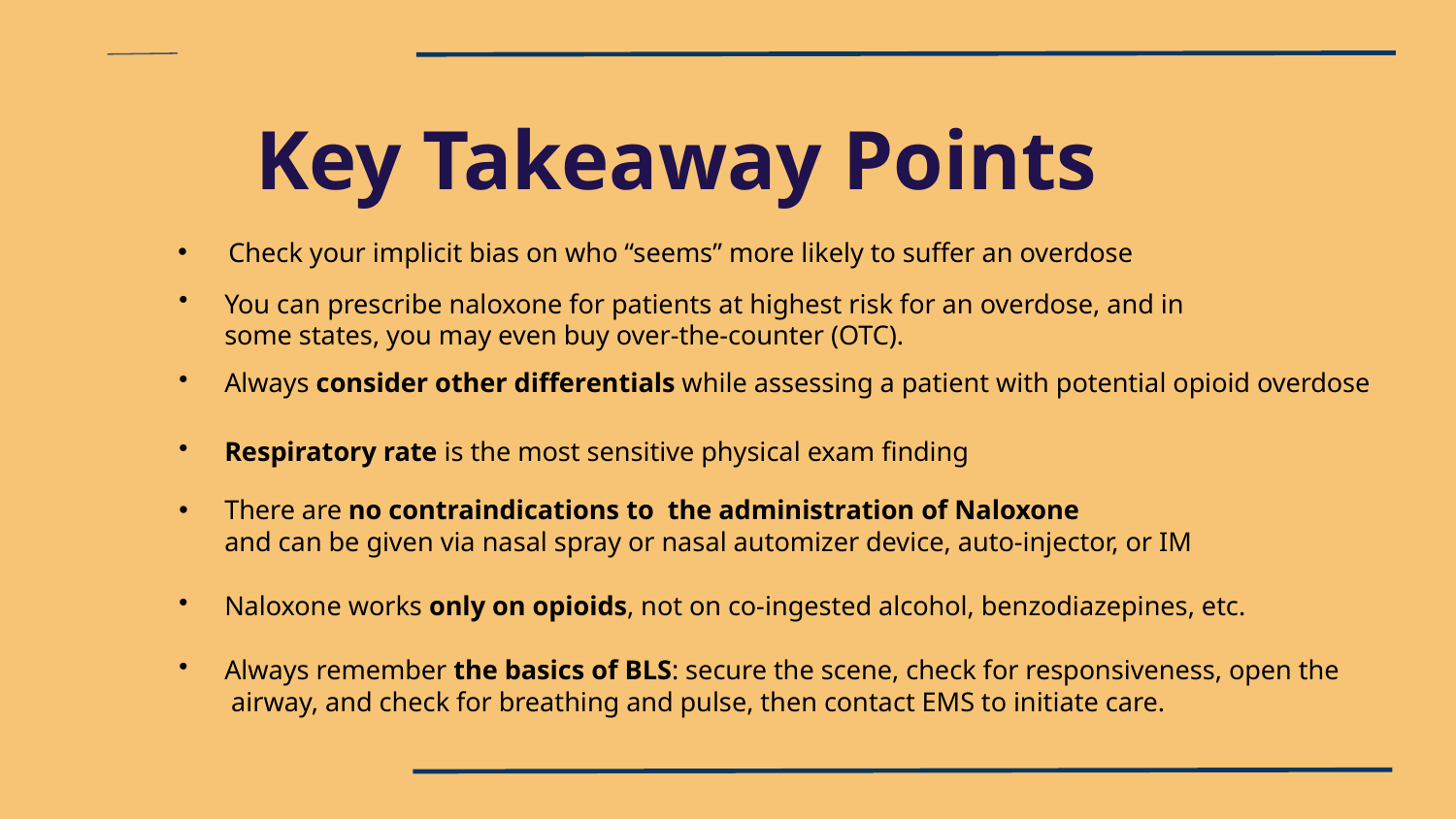

Key Takeaway Points
Check your implicit bias on who “seems” more likely to suffer an overdose
You can prescribe naloxone for patients at highest risk for an overdose, and in some states, you may even buy over-the-counter (OTC).​
Always consider other differentials while assessing a patient with potential opioid overdose​​
Respiratory rate is the most sensitive physical exam finding​​​
There are no contraindications to  the administration of Naloxone and can be given via nasal spray or nasal automizer device, auto-injector, or IM
Naloxone works only on opioids, not on co-ingested alcohol, benzodiazepines, etc. ​​​​​
Always remember the basics of BLS: secure the scene, check for responsiveness, open the airway, and check for breathing and pulse, then contact EMS to initiate care.​​

## Slide 15
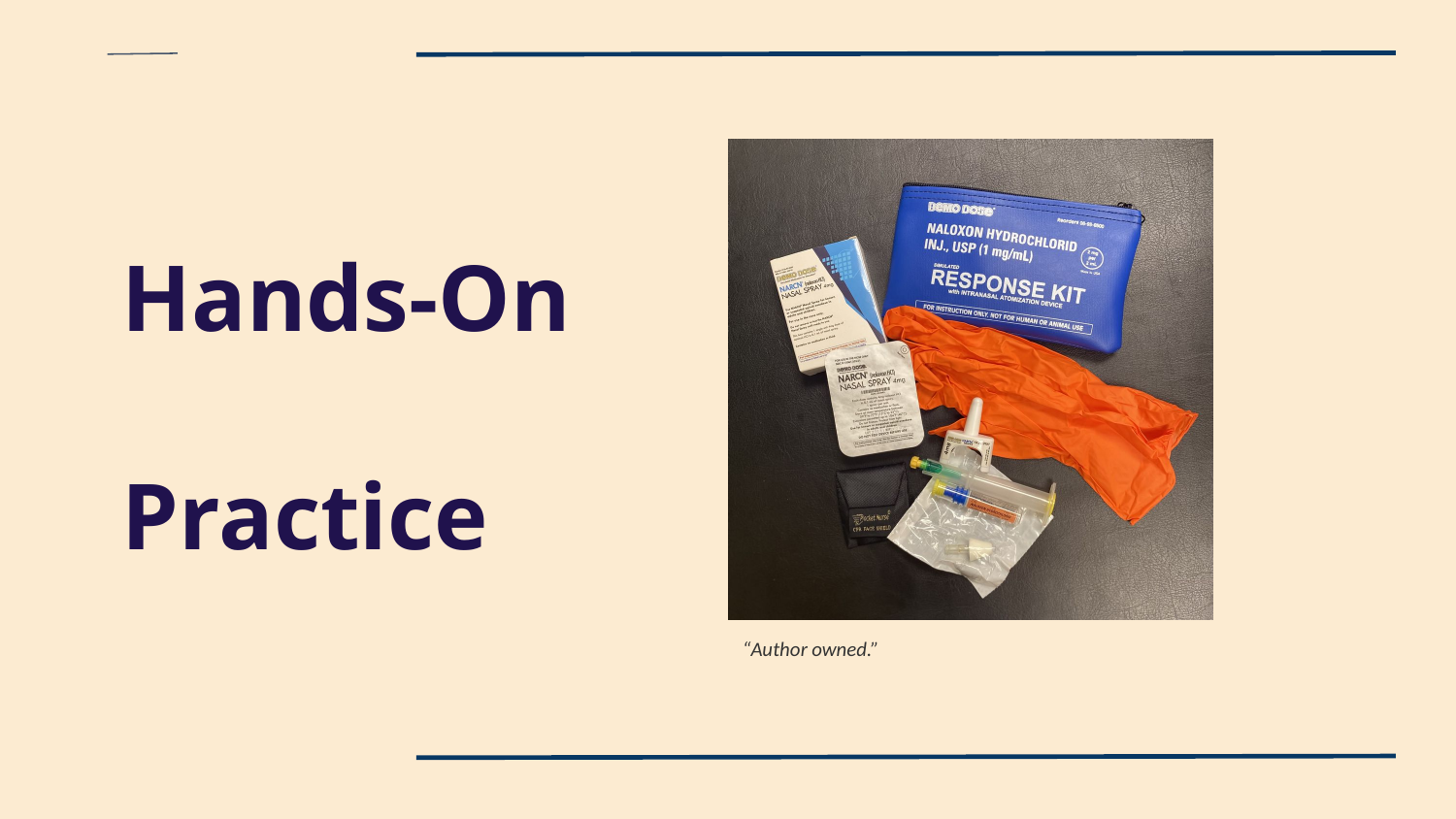

Hands-On
Practice
“Author owned.”

## Slide 16
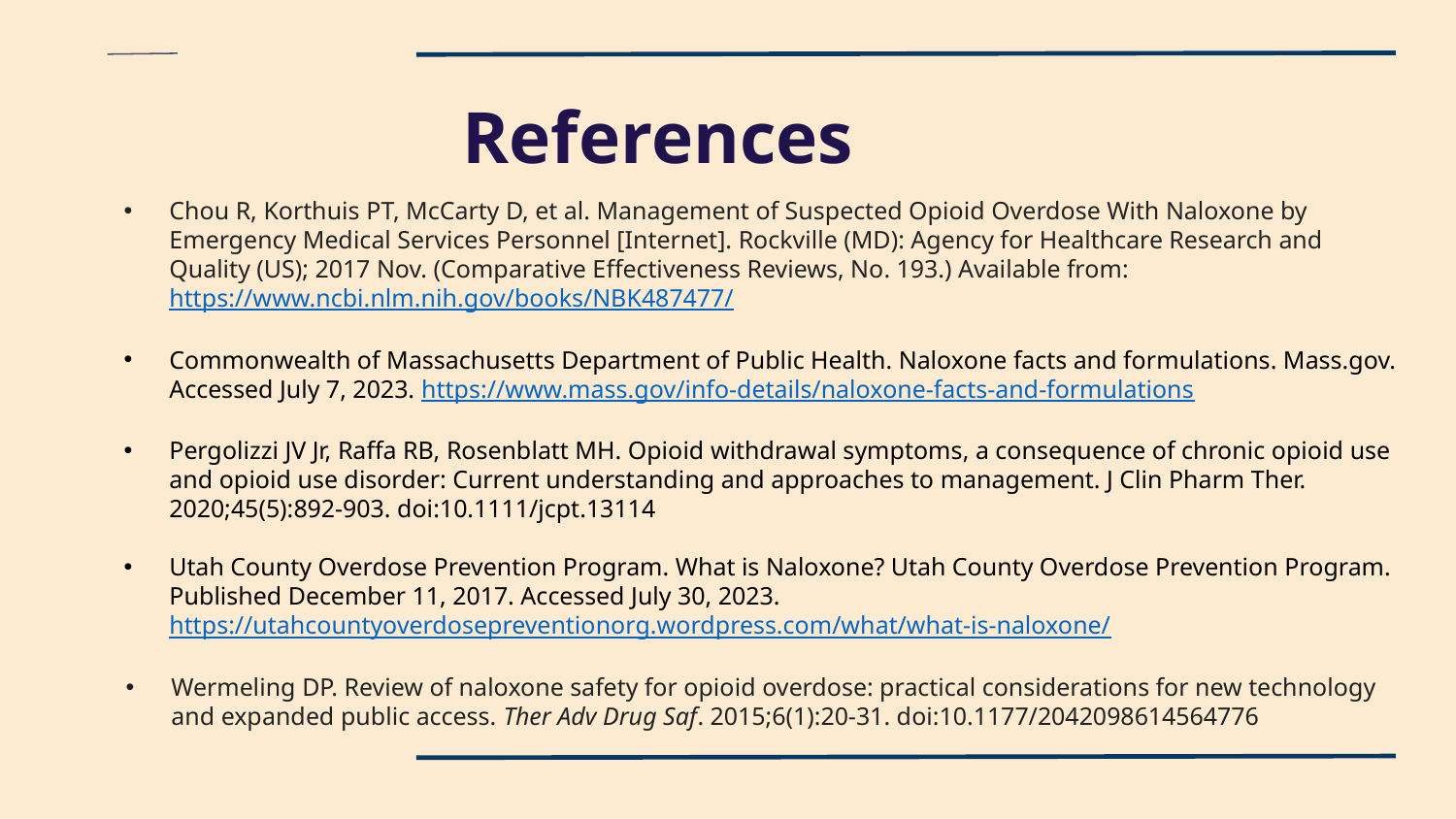

References
Chou R, Korthuis PT, McCarty D, et al. Management of Suspected Opioid Overdose With Naloxone by Emergency Medical Services Personnel [Internet]. Rockville (MD): Agency for Healthcare Research and Quality (US); 2017 Nov. (Comparative Effectiveness Reviews, No. 193.) Available from: https://www.ncbi.nlm.nih.gov/books/NBK487477/
Commonwealth of Massachusetts Department of Public Health. Naloxone facts and formulations. Mass.gov. Accessed July 7, 2023. https://www.mass.gov/info-details/naloxone-facts-and-formulations
Pergolizzi JV Jr, Raffa RB, Rosenblatt MH. Opioid withdrawal symptoms, a consequence of chronic opioid use and opioid use disorder: Current understanding and approaches to management. J Clin Pharm Ther. 2020;45(5):892-903. doi:10.1111/jcpt.13114
Utah County Overdose Prevention Program. What is Naloxone? Utah County Overdose Prevention Program. Published December 11, 2017. Accessed July 30, 2023. https://utahcountyoverdosepreventionorg.wordpress.com/what/what-is-naloxone/
Wermeling DP. Review of naloxone safety for opioid overdose: practical considerations for new technology and expanded public access. Ther Adv Drug Saf. 2015;6(1):20-31. doi:10.1177/2042098614564776

## Slide 17
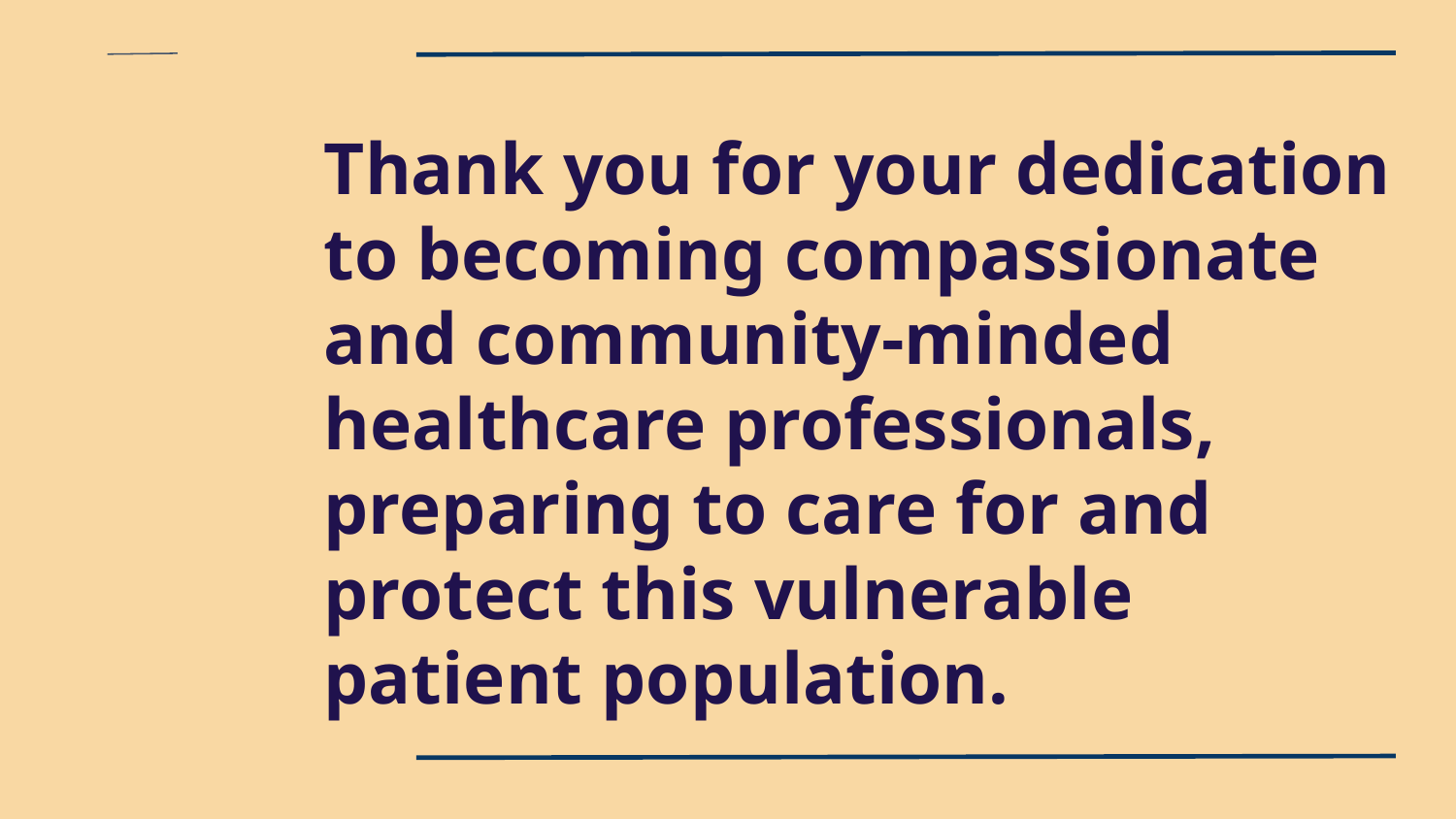

Thank you for your dedication to becoming compassionate and community-minded healthcare professionals, preparing to care for and protect this vulnerable patient population.

## Slide 18
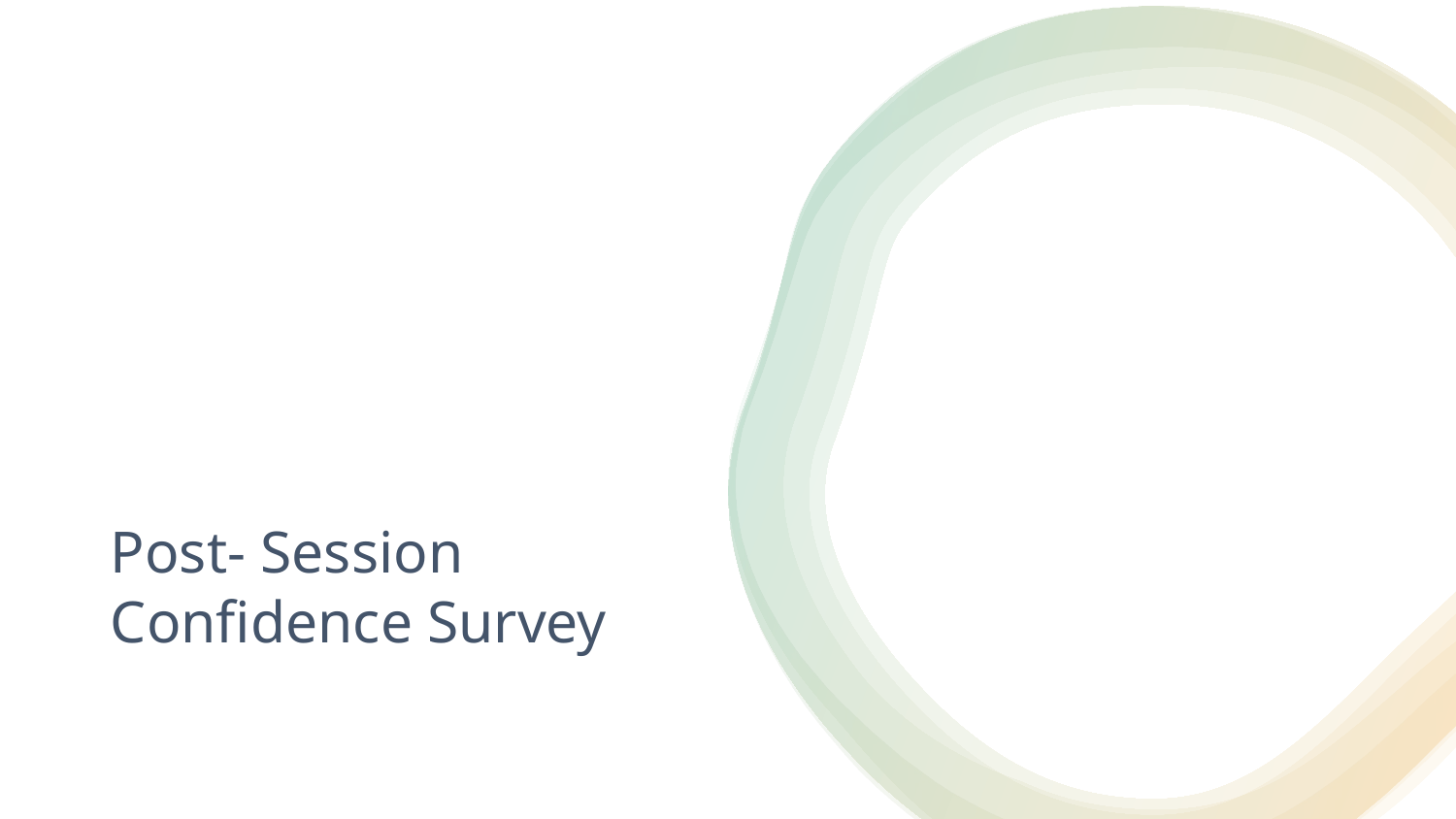

# Post- Session Confidence Survey
