# Supplementary material for: Empowering Future Physicians: Enhancing Naloxone Competency Through Early Harm Reduction Training in Medical Education
Source: MedEdPORTAL. 2025 Feb 14;21:11499. doi: 10.15766/mep_2374-8265.11499 (PMC11825861; doi:10.15766/mep_2374-8265.11499)
Supplement: Supplementary file 1 — Facilitator Guide.docxOpioid Overdose Statistics Lecture.pptxHarm Reduction Initiatives Lecture.pptxCase-Based Discussion Scenario.pptxOSCE-Style Checklist.docxTraining Session Confidence Survey.docx [file mep_2374-8265.11499-s001.zip › E. OSCE-Style Checklist.docx]

**Appendix E: OSCE-Style Checklist for Hands-On Practice**

| **Skills Check for Administering Naloxone Nasal Spray** | **Done** | **Done incorrectly** | **Not Done** |
| --- | --- | --- | --- |
| Verbalizes assessment of scene safety before approaching patient |  |  |  |
| Checks for patient responsiveness verbally, then via firm shaking of patient’s shoulder/arm or via sternal rub |  |  |  |
| Calls/ instructs bystander to call 911 |  |  |  |
| Checks patient’s pulse (5-10 sec) and begins CRP if pulseless |  |  |  |
| **Student is told that patient’s pulse is weak but present, and that needles and drug paraphernalia are seen lying beside them** | | | |
| Confirms suspected opiate overdose with proper indications for treatment (altered mentation, respiration compromise, pinpoint pupils). Only 1 indication required. |  |  |  |
| Accesses Naloxone Training Kit, student can complete either A or B, only one of these tasks is required | | | |
| 1. **Obtains and correctly administers (Narcan ®) nasal spray following steps below:** | | |  |
| 1. Lay the person of their back to administer Naloxone |  |  |  |
| 1. Remove medication from box. Peel back the tab with a circle to open |  |  |  |
| 1. Hold the Naloxone spray with thumb on bottom of plunger and the third and middle fingers on the either side of the nozzle. |  |  |  |
| 1. Tilt the person’s head back. Support the head with your hand under the neck |  |  |  |
| 1. Gently insert the tip of the nozzle until your fingers on either side of the nostril are against the bottom of the person’s nose |  |  |  |
| 1. Press the plunger firmly to give the entire dose to one nostril |  |  |  |
| 1. Remove naloxone nasal spray and place patient in recovery position (on their side) |  |  |  |
| 1. Monitor person, administer rescue breathing if needed. If no response to talking, touch or pain, additional Naloxone nasal spray may be administered every 2-3 minutes if available, until the person responds, or EMS arrives. |  |  |  |
| 1. If needed, use steps 2-8 using a new Narcan nasal spray in the other nostril |  |  |  |
| 1. **Obtains and correctly administers Naloxone nasal spray following steps below:** | | |  |
| 1. Lay the person on their back to administer Naloxone |  |  |  |
| 1. Remove syringe and vial from properly labeled box |  |  |  |
| 1. Removes both yellow caps from end of syringe |  |  |  |
| 1. Connect atomizer tip to syringe |  |  |  |
| 1. Gently screws vial of naloxone clockwise into the barrel the large end of the syringe |  |  |  |
| 1. Tilt the person’s head back. Support the head with your hand under the neck |  |  |  |
| 1. Place atomizer 1.5 cm into one nostril and briskly compress the syringe to administer half (1 cc) of the medication. |  |  |  |
| 1. Move atomizer to 2^nd^ nostril and repeat; syringe should be completely empty |  |  |  |
| 1. Monitor person, administer rescue breathing if needed. If no response to talking, touch or pain, additional Naloxone nasal spray may be administered every 2-3 minutes if available, until the person responds, or EMS arrives. |  |  |  |
| 1. If needed, use steps 2-8 using a new Naloxone nasal spray in the other nostril |  |  |  |
| 1. Place patient in recovery position (on their side) |  |  |  |
| **Student is told that patient is stirring and EMS has just arrived to transport patient to hospital** | | | |
