# Supplementary material for: Empowering Future Physicians: Enhancing Naloxone Competency Through Early Harm Reduction Training in Medical Education
Source: MedEdPORTAL. 2025 Feb 14;21:11499. doi: 10.15766/mep_2374-8265.11499 (PMC11825861; doi:10.15766/mep_2374-8265.11499)
Supplement: Supplementary file 1 — Facilitator Guide.docxOpioid Overdose Statistics Lecture.pptxHarm Reduction Initiatives Lecture.pptxCase-Based Discussion Scenario.pptxOSCE-Style Checklist.docxTraining Session Confidence Survey.docx [file mep_2374-8265.11499-s001.zip › F. Training Session Confidence Survey.docx]

**Appendix F. Training Session Confidence Survey**

**Question 1:**

I am confident in my ability to assess a patient with potential opioid overdose.

**Answer Choices:**

A. Strongly Agree

B. Agree

C. Neither Agree nor Disagree

D. Disagree

E. Strongly Disagree

**Question 2:**

I am confident in my ability to administer Naloxone to a patient with suspected opioid overdose.

**Answer Choices:**

A. Strongly Agree

B. Agree

C. Neither Agree nor Disagree

D. Disagree

E. Strongly Disagree

**Question 3:**

I am confident in my ability to continue the management of a patient with opioid overdose, after Naloxone has been administered.

**Answer Choices:**

A. Strongly Agree

B. Agree

C. Neither Agree nor Disagree

D. Disagree

E. Strongly Disagree

**Question 4:**

I am confident in my ability to train others on Naloxone use.

**Answer Choices:**

A. Strongly Agree

B. Agree

C. Neither Agree nor Disagree

D. Disagree

E. Strongly Disagree
