# Supplementary material for: Empowering Future Physicians: Enhancing Naloxone Competency Through Early Harm Reduction Training in Medical Education
Source: MedEdPORTAL. 2025 Feb 14;21:11499. doi: 10.15766/mep_2374-8265.11499 (PMC11825861; doi:10.15766/mep_2374-8265.11499)
Supplement: Supplementary file 1 — Facilitator Guide.docxOpioid Overdose Statistics Lecture.pptxHarm Reduction Initiatives Lecture.pptxCase-Based Discussion Scenario.pptxOSCE-Style Checklist.docxTraining Session Confidence Survey.docx [file mep_2374-8265.11499-s001.zip › A. Facilitator Guide.docx]

## **Facilitator Guide: Opioid Overdose Response Training Session**

**Schedule and Brief Agenda**

1. Introduction and Pre-training Survey (5 min)
2. Lecture on Opioid Crisis and Opioid Use Disorder Statistics (25 min)
3. Community Outreach Member Presentation on Harm Reduction Strategies. (20 min)
4. Opioid Overdose Response Training (60 min)
5. Post-Training Survey and Session Wrap-Up (10 min)

**Overview:** This guide outlines the flow of the Opioid Overdose Response Training session, including the timing and use of appendices, as well as sourcing and incorporating community outreach members. The training is designed to equip medical students with the skills and knowledge to recognize and respond to opioid overdoses using naloxone.

**Learning Objectives:**

1. Identify the signs of an opioid overdose (Bloom’s level 2 – understand)
2. Describe the mechanism of action of naloxone (level 2 - understand)
3. Compare and contrast the different formulations of naloxone preparations (level 4 - analyze)
4. Describe the routes by which the different naloxone formulations are administered to patients (level 2 - understand)
5. Demonstrate how to administer naloxone to patients (level 3 - apply)

The following are essential materials and personnel for effective hands-on training:

- **1 faculty facilitator** and **3 senior medical student trainers** per group
- **1 Community Outreach member** facilitator
- **1 mannequin** per 3-4 students- task trainer used for Cardiopulmonary Resuscitation (CPR) with/without pinpoint pupils
- **Opioid Overdose Prevention Kit**, which includes:
  - 1 Luer-Jet 2mg/2mL prefilled syringe
  - 1 nasal atomizer
  - 1 nasal spray device
  - 1 pair of gloves
  - 1 CPR face shield

### **Session Flow**

**1. Introduction and Pre-Training Survey**

- **Duration:** 5 minutes
- **Activity:**
  - If planning to formally evaluate change in knowledge, instruct students to complete the voluntary pre-training survey via QR code using their mobile phones.
  - **Materials:**
    - **Appendix F**: Training Session Confidence Survey
- **Instructions:**
  - Ensure all students have access to the QR code and understand the survey's purpose.

**2. Lecture on Opioid Crisis and Opioid Use Disorder Statistics**

- **Duration:** 25 minutes
- **Activity:**
  - Present an overview of the current national opioid crisis, statistics on opioid use disorder, and harm reduction strategies.
- **Materials:**
  - Appendix B: Opioid Overdose Statistics Lecture
- **Instructions:**
  - Describe the significance of opioid-related deaths as a leading cause of preventable deaths and reference sources such as the Centers for Disease Control’s National Vital Statistics Reports for the most updated statistics. Introduce harm reduction as a framework or approach to caring for individuals and families facing substance use disorders. Reference the Substance Abuse and Mental Health Services Administration’s “Harm Reduction Framework.” Emphasize the importance of harm reduction strategies like naloxone administration to prevent opioid-related deaths.

**3. Community Outreach Member Presentation and Harm Reduction Strategies**

- **Duration:** 20 minutes
- **Activity:**
  - A community outreach member shares their personal experience with opioid use disorder and discusses current community initiatives, such as syringe exchange programs and naloxone distribution centers.
- **Materials**:
  - Appendix C: Harm Reduction Initiatives Lecture
- **Instructions for Sourcing:**
  - **Sourcing Community Outreach Members:**
    - Collaborate with local community health organizations, recovery centers, or harm reduction programs to identify individuals with lived experience in opioid use disorder.
    - Consider individuals who are already involved in outreach or educational activities within the community.
    - Ensure the selected individual is comfortable sharing their story and can effectively communicate the importance of harm reduction efforts.
  - **Preparation:**
    - Schedule a briefing with the outreach member to align their presentation with the session's objectives.
    - Provide them with an overview of the student audience and the context of the session.

**4. Opioid Overdose Response Training**

- **Duration:** 60 minutes
- **Activity:**
  - Lead the case-based discussion on opioid overdose recognition and naloxone administration.
- **Materials:**
  - **Appendix D:** Case-Based Discussion Scenario
  - **Appendix E:** OSCE-Style Checklist for Hands-On Practice
- **Instructions:** This section includes:
  - **Case-Based Discussion** (20 minutes):

Ask a student to read the patient scenario depicting a suspected opioid overdose. Then, pose the case questions to prompt discussion of how to approach the evaluation and management of an opioid overdose. Emphasize the clinical signs of an opioid overdose.

- - **Naloxone Administration Demonstration** (15 minutes):

Review the basic pharmacology of naloxone as outlined in the slide presentation. Present the different formulations of naloxone and describe the proper assembly and use of naloxone delivery systems.

- - **Interactive Hands-On Practice** (25 minutes):

Allow students to practice overdose recognition and naloxone administration using task trainers. Divide students into groups of 3-5 and distribute the OSCE-style checklist. Ask them to use the OSCE-style checklist as they approach the evaluation of a suspected overdose. They should take turns practicing assessing for response and breathing as well as delivering naloxone.

**5. Post-Training Survey and Session Wrap-Up**

- **Duration:** 10 minutes
- **Activity:**
  - Students complete the post-training survey to evaluate the session’s impact.
  - **Materials:**
    - **Appendix F:** Training Survey
- **Instructions:**
  - Direct students to complete the post-training survey using the provided QR code.

**Conclusion:** Conclude the session by summarizing key takeaways and emphasizing the importance of continued learning and community involvement in opioid overdose prevention.

### **Appendix Usage Summary**

- **Appendix A:** Facilitator Guide – Use to guide the session, including the timing and use of appendices
- **Appendix B:** Opioid Overdose Statistics Lecture - structured way to deliver the session's content
- **Appendix C:** Harm Reduction Initiatives Lecture - offers a clear structure for presenting the session's content, ensuring it is both informative and engaging.
- **Appendix D:** Case-Based Discussion Scenario – Utilized during the Opioid Overdose Response Training for the case-based discussion.
- **Appendix E:** OSCE-Style Checklist – Provided to students during the hands-on practice activity for guided practice.
- **Appendix F:** Training Session Confidence Survey – Used at the start of the session during the Introduction and once again completed by students at the end of the session to evaluate training impact.

### **Additional Notes for Facilitators**

- **Community Engagement:** Establish ongoing relationships with local community organizations to maintain a pool of potential outreach members for future sessions.
- **Session Timing:** Adhere to the session timeline to ensure all activities are completed within the allocated two-hour timeframe.
- **Student Support:** Be available to answer questions and provide additional guidance during hands-on practice to reinforce learning.

This facilitator guide is intended to provide structure and support for delivering a comprehensive and impactful Opioid Overdose Response Training session, ensuring medical students are well-prepared to handle opioid-related emergencies before entering their clinical years (Year 3 and Year 4).
