# Supplementary material for: Empowering Future Physicians: Enhancing Naloxone Competency Through Early Harm Reduction Training in Medical Education
Source: MedEdPORTAL. 2025 Feb 14;21:11499. doi: 10.15766/mep_2374-8265.11499 (PMC11825861; doi:10.15766/mep_2374-8265.11499)
Supplement: Supplementary file 1 — Facilitator Guide.docxOpioid Overdose Statistics Lecture.pptxHarm Reduction Initiatives Lecture.pptxCase-Based Discussion Scenario.pptxOSCE-Style Checklist.docxTraining Session Confidence Survey.docx [file mep_2374-8265.11499-s001.zip › _Educational Summary Report_11499.pdf]

MedEdPORTAL® The AAMC Journal of Teaching and Learning Resources

Original Publication Open Access

Empowering Future Physicians: Enhancing Naloxone Competency Through Early Harm Reduction Training in Medical Education

Alexa Ovalles Lacruz*, Maria S. Valle, Karla Santoyo, Rachel D. Clarke, PhD, CHES, Prasad Bhoite, MBA, MPH, MSc, Lizzeth N. Alarcon, MD

*Corresponding author: aoval008@med.fiu.edu

Abstract

Introduction: With increasing rates of fatal opioid overdoses and substance use disorders, discussing harm reduction strategies in undergraduate medical education is imperative. Medical students learn opioid pharmacology but often lack adequate training in recognizing and responding to opioid overdoses. Incorporating naloxone training into preclinical undergraduate medical education can be lifesaving. **Methods:** This educational activity trained second-year medical students in opioid overdose recognition and response. The 2-hour interactive session included an informative presentation on opioid use statistics, harm reduction, and opioid overdose; a case-based learning session; and hands-on practice on task trainers using an OSCE-style checklist. Student confidence in four areas was assessed through pre- and posttraining surveys on 5-point Likert scales. Data analysis was conducted using R (programming language), with exploratory analyses for sample size, normality, and variable selection. The Wilcoxon signed rank test evaluated changes in attitudes from pre- to posttest. **Results:** Eighty-six students participated, with 60 completing pre- and posttest surveys. Significant improvements were observed in confidence across all areas: assessing an opioid overdose ($p < .001$), administering naloxone ($p < .001$), continuing management after naloxone ($p < .001$), and training others on naloxone administration ($p < .001$). **Discussion:** Early training in opioid overdose management increased second-year medical students' confidence in key areas, potentially enabling them to better combat the crisis during their clinical years and educate at-risk patients. Future activities should incorporate objective skill assessments to evaluate competency and explore long-term knowledge retention during clinical rotations.

Keywords
Harm Reduction, Naloxone, Opioid Overdose Prevention, Substance Use Disorders, Case-Based Learning, Opioids, Addiction, & Pain, Preventive Medicine, Community Engagement, Community-Engaged Learning

Educational Objectives

By the end of this activity, learners will be able to:

1. Identify the signs of an opioid overdose.
2. Describe the mechanism of action of naloxone.
3. Compare and contrast the different formulations of naloxone preparations.
4. Describe the routes by which the different naloxone formulations are administered to patients.
5. Demonstrate how to administer naloxone to patients.

Citation:
Ovalles Lacruz A, Valle MS, Santoyo K, Clarke RD, Bhoite P, Alarcon LN. Empowering future physicians: enhancing naloxone competency through early harm reduction training in medical education. *MedEdPORTAL*. 2025;21:11499.
https://doi.org/10.15766/mep_2374-8265.11499

Introduction

As the global drug crisis escalates and opioid overdose remains a leading cause of accidental death in the US,[1] medical schools are integrating more content on substance use disorders (SUDs), particularly opioid use disorder.[2] The opioid epidemic continues to be a major public health crisis in the US.[3] While opioid prescribing rates have declined, overdose deaths involving synthetic opioids have risen dramatically.[1] In 2022, there were 107,941 drug-involved overdose deaths reported in the US.[1] More specifically, opioid-involved overdose deaths rose from 49,860 in 2019 to 81,806 in 2022.[1]

The World Health Organization recommends training anyone likely to witness an overdose on using opioid reversal agents and managing overdoses in order to save lives[4]; however, these strategies are often stigmatized due to their association with drug use and addiction.[5] In addition to perceived stigma, there is self-stigmatization, which occurs among the patients themselves,

Copyright © 2025 Ovalles Lacruz et al. This is an open-access publication distributed under the terms of the Creative Commons Attribution license.

pharmacists, and prescribers.[5] Implementing training for health care professionals has shown increased opioid overdose competency and willingness to use naloxone.[6,7] With rising rates of overdose and SUDs, discussing harm reduction strategies early in medical training may enhance students' knowledge and attitudes. Educational interventions can play a crucial role by equipping health care students with the knowledge and skills to identify and respond to opioid-related health needs. Previous initiatives have shown that training during medical school presents an opportunity to reduce SUD stigma and improve opioid overdose reversal knowledge.[2,8] Interprofessional naloxone administration trainings including nursing, physician assistant, and pharmacy students resulted in increased knowledge, confidence, and interprofessional teamwork as well as greater skills in counseling patients regarding SUD.[9,10]

Medical students, who typically possess foundational knowledge of opioid pharmacology, report barriers to naloxone administration, including lack of hands-on experience and confidence in emergency overdose interventions.[11] This gap in skills and attitudes towards opioid overdose management necessitates a more comprehensive educational approach. Incorporating more SUD and harm reduction content into undergraduate medical education during the preclinical years can be lifesaving. At this level of training, medical students can enter their required clerkships with the knowledge to identify those individuals battling addiction and at high risk of overdose. We examined the changes in student knowledge and confidence following the integration of an opioid overdose reversal training into a required course on social determinants of health and health equity at a large single-campus medical school in South Florida. By addressing the critical need for improved overdose management training and leveraging a comprehensive instructional approach, this training initiative offers a significant advancement in medical education, with the potential to be adapted and implemented across various medical schools and other health professions' training programs.

Florida International University Herbert Wertheim College of Medicine is a community-based medical school whose curriculum emphasizes community health and the social determinants of health. Although students receive general instruction on the pharmacology of opioids and identification and management of SUDs, curricular activities focusing on harm reduction strategies in this undergraduate medical education program are limited. Student interest in improving medical knowledge about harm reduction led to the design of this training. Students developed this curricular activity under the guidance of the clinical skills simulation center faculty advisor and served as the facilitators for the training.

Prior *MedEdPORTAL* publications include several training initiatives on opioid management in the inpatient setting as well as the emergency department setting using standardized patients and case-based scenarios. However, most of these initiatives are aimed at residents and pharmacy students.[12-15] There is one program using first-year medical students who complete a case-based learning exercise to understand the pharmacological principle behind opioids.[16] The educational initiative we conducted, approved by our institutional review board, is one of the first to incorporate different learning methods—presentation on harm reduction strategies and the opioid crisis, a case-based learning session, and hands-on practice with task trainers—using preclinical medical students. While other educational approaches on opioid management exist, they do not tailor the training to preclinical medical students and do not include hands-on practice with task trainers in combination with access to a real opioid overdose prevention kit. By combining presentations, a case-based learning session, and hands-on practice with a detailed checklist, we offer a comprehensive and practical approach to opioid overdose prevention. The OSCE-style checklist for the hands-on opioid overdose scenario ensures consistency and effectiveness in the teaching process. By guiding the evaluation of students' performance, the checklist helps to maximize the learning experience. Additionally, our training initiative's unique feature of including a community outreach member sharing personal experiences with opioid use disorder and discussing local initiatives like syringe exchange programs and naloxone distribution centers provides a valuable real-world perspective. This element, not commonly found in other literature, supports the relevance and applicability of our training to students' future medical practice.

Methods

Content and Delivery

This educational session was designed to provide preclinical medical students with essential skills in opioid overdose management and naloxone administration. Integrated into the students' Community Engaged Physician course, which covered interprofessional teamwork, population health, and social determinants of health, the training took place during a lecture focused on injury and violence prevention. The facilitator guide (Appendix A) outlined the overall flow of the activity and provided a detailed framework for conducting the opioid overdose response training session.

Copyright © 2025 Ovalles Lacruz et al. This is an open-access publication distributed under the terms of the Creative Commons Attribution license.

This session included four key components: (1) a lecture overview of the opioid crisis and opioid use disorder statistics (Appendix B), (2) a presentation on harm reduction strategies (Appendix C), (3) a case-based discussion with naloxone administration demonstration (Appendix D), and (4) an interactive, hands-on practice activity on overdose recognition and naloxone administration using task trainers and an OSCE-style checklist (Appendix E). A confidence survey was administered pre- and posttraining (Appendix F).

A faculty member presented a lecture that included recent statistics on opioid use and overdose, followed by a community outreach member who shared personal experiences with opioid use disorder and provided information on local harm reduction resources, such as syringe exchange programs and naloxone distribution centers. The case-based learning component featured a simulated patient scenario involving a suspected opioid overdose, guiding students through clinical signs and assessment of the scene. Students were introduced to different naloxone delivery systems (injectable, auto-injectable, intranasal spray, and nasal atomizer) and practiced assembly using a demonstration kit. For the hands-on component, students worked in groups of three to five with a task trainer, an OSCE-style checklist (Appendix E), and an opioid overdose prevention kit, enabling them to practice key steps such as scene assessment, clinical sign recognition, and naloxone administration. The kits were funded by a grant through the Opioid Response Network (Figure). We utilized an OSCE-style framework since it guided the students through specific steps needed to complete the practice activity[17] and the students used this framework for their other practice-based activities in the curriculum. The same clinical staff who developed the OSCE examinations for our medical school's clinical simulation center structured this checklist. We designed the OSCE checklist to pair with the simulated patient scenario and utilized first-response guidelines and naloxone administration instructions. The students practiced assessment of scene safety, voicing recognition of clinical signs of opioid-related overdose, checking responsiveness, and instructing bystanders to call for help, followed by proper assembly of the nasal atomizer and delivery of naloxone nasal spray.

The session incorporated experiential learning by allowing students to apply theoretical knowledge in a practical setting, thereby enhancing motivation and retention.[18] Students completed a pre- and posttraining survey (Appendix F) to evaluate the session's impact, with no incentives for participation. The entire activity lasted approximately 2 hours, with the opioid overdose response training itself taking around 1 hour. This

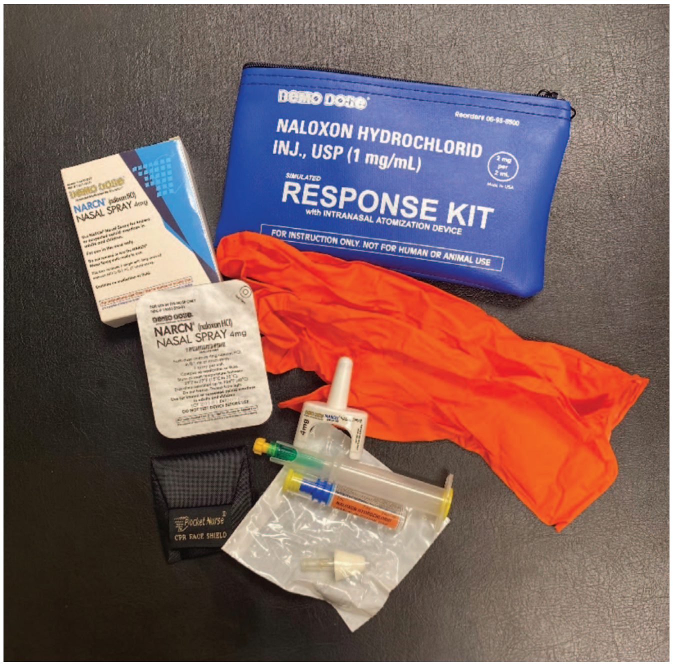

Figure. Contents of an overdose prevention kit. Each kit contains one Luer-Jet 2mg/2mL prefilled syringe, one nasal atomizer, one nasal spray device, one pair of gloves, and a CPR face shield. Photo: author owned.

approach offered a comprehensive method for equipping future health care professionals with vital skills in overdose management and naloxone administration, adaptable to various health professions' training programs.

Student Participants and Exclusion Criteria

The target audience for this educational activity consisted of preclinical medical students in their first or second year of medical school. As preclinical students, they had not yet gained hands-on experience in clinical clerkships, making this training particularly valuable. By focusing on students who were early in their medical education, the training provided foundational knowledge and skills in opioid overdose recognition and response before the students entered clinical settings. This early exposure aimed to build confidence and competence in managing opioid-related emergencies, which participants would later encounter in real-world clinical environments.

A total of 120 second-year medical students were present for the training. The training occurred during their required Community Engaged Physician course. Eighty-six students completed the pretraining survey, and 60 students completed the posttraining surveys. Students who had received naloxone training during the development sessions in the months leading up to this current training were allowed to participate in the activity but

Copyright © 2025 Ovalles Lacruz et al. This is an open-access publication distributed under the terms of the Creative Commons Attribution license.

were excluded from completing the pre- and postsurveys. Students who completed the presession survey but failed to complete the postsession survey were excluded from the data analysis. Duplicate or incomplete student identifications were also excluded from the data analysis.

Evaluation Strategy

Survey: We evaluated the session's impact by administering pre- and postsession questionnaires. The key areas we measured were students' change in confidence assessing and responding to an opioid overdose and their ability to train others in naloxone use. The pretraining survey consisted of two identifying questions to link individual pre- and posttraining survey responses, followed by four questions about self-perceived confidence in the recognition and management of opioid overdose and reversal. The posttraining survey contained identical questions. A Likert-type scoring scale was the preferred option as it was user-friendly and easily understood, as well as providing acceptable levels of reliability to measure attitudes, beliefs, and opinions.[19] To measure confidence appropriately, the attitude questions were scored on a 5-point scale (1 = *strongly disagree,* 2 = *disagree,* 3 = *neither agree nor disagree,* 4 = *agree,* 5 = *strongly agree*).

Data collection and analysis: We collected all pre- and posttraining survey responses electronically using the web-based software Qualtrics. Responses for both questionaries were exported, and all data were deidentified before analysis. R programming language version 4.3.1 was used to analyze quantitative data. We assessed exploratory analyses of sample size, normality, and variable selection. We used the Wilcoxon signed rank test to assess changes in attitudes from pre- to posttest. Changes were considered significant at the alpha level of .05.

Results

One hundred twenty second-year medical students were present for the training. Eighty-six completed the pretraining survey, and 60 completed the posttraining surveys, for a 50% response rate. There were statistically significant improvements in the mean for all four confidence parameters from the pre- to posttraining confidence surveys, with p values of <.001. The four competency-based goals, as seen in the Table, focused on the assessment of opioid overdose, confidence with naloxone administration, management after administration of naloxone, and confidence in training others on the use of naloxone.

Presurvey results revealed most students did not feel confident with assessing opioid overdose patients (15%) and administering naloxone (2%), while postsurvey results showed a significant increase to 93% and 60%, respectively. Confidence in postadministration management of an opioid overdose patient also increased significantly. The final parameter, addressing confidence in training others on naloxone administration, showed a shift from 77% disagreeing presurvey to 90% agreeing postsurvey. The Table highlights the significant improvements in participant confidence following the educational activity.

Table. Pre- and Posttraining Survey Confidence Results (N = 60)

	***M* (*SD*)**		
**Confidence Area**[a]	**Pretraining Survey**	**Posttraining Survey**	***p***
Assessment of an opioid overdose	2.6 (0.8)	4.5 (0.6)	<.001
Administration of naloxone	2.4 (0.9)	4.6 (0.6)	<.001
Management after administration of naloxone	2.2 (0.8)	4.4 (0.7)	<.001
Training others on the use of naloxone	2.1 (0.9)	4.4 (0.7)	<.001

[a]Rated on a 5-point Likert scale (1= *strongly disagree,* 5 = *strongly agree*).

Discussion

After the completion of the opioid reversal training session, participating second-year medical students were able to identify the signs and symptoms of an opioid overdose, describe the mechanism of action of naloxone, compare and contrast the different formulations of naloxone preparations, demonstrate how to administer naloxone to patients, and explain how naloxone can save the life of patients in an opioid overdose. Through our pre- and postquestionnaire evaluation data, we observed a significant increase in students' self-reported confidence across four parameters (identifying, administering, managing, and training on naloxone), which suggests our training session effectively addressed its learning objectives.

This educational activity's goal was to measure the change in confidence related to opioid overdose management immediately following a curricular naloxone training integrated into a preclinical class session. Our resource represents a unique contribution that incorporates a blend of theoretical knowledge and practical skills in comprehensive harm reduction in undergraduate medical education. Previous interventions have shown that training for naloxone use across various groups (emergency medical technicians, pharmacists, and clinicians) improves competency and confidence in handling opioid overdoses.[6,7,10] This can reduce mortality and improve patient safety. Early exposure to SUD topics during medical training can mitigate the negative stigma around SUD.[20] Harm reduction education increases overdose knowledge and confidence in addressing opioid overdose.[21] Nationwide, medical schools are

Copyright © 2025 Ovalles Lacruz et al. This is an open-access publication distributed under the terms of the Creative Commons Attribution license.

adopting a stepwise approach to enhance SUD education by incorporating targeted training programs, but harm reduction curricula and teaching of harm reduction principles are still lacking.[22] Our didactic educational session contributes to these efforts with an intentional focus on harm reduction education in the preclinical years.

Collaborating with clinical skills faculty and utilizing readily available resources ensured cost-effectiveness and facilitated future program replication. In addition, this structured approach could be applied to develop simulations for high-risk clinical scenarios, such as handling cardiac arrests, managing sepsis, or performing trauma assessments. Incorporating hands-on practice with clear curricular context, content delivery, and criteria for participation ensures students are well prepared for real-life emergencies. Our session was implemented during a single academic year, and we have gained valuable insights from this initial experience. One challenge we encountered was ensuring that the training was engaging and effectively integrated with the broader curriculum on the social determinants of health. To address this, we collaborated with community partners to make the content more relevant and relatable, allowing students to connect the training to real-world applications. Based on participant feedback, we also recognized the importance of reinforcing these skills over time. Moving forward, we plan to expand the training, incorporating periodic assessments and interactive sessions to build on the foundational knowledge provided in the preclinical years.

Given the success of our didactive educational activity, we are advocating for our school to incorporate it into the second-year curriculum for medical students before starting their clerkship requirements. Ensuring all preclinical students receive this training as a curricular experience is a proactive approach and encourages all students to take an active role during their clerkships when caring for patients with SUDs. We are also exploring ways to incorporate follow-up sessions during clerkship years to strengthen students' confidence and skills in managing SUD cases.

There are limitations to our educational activity. First, we measured self-reported data assessing confidence in recognizing and responding to an opioid overdose. Although we used the OSCE checklists to guide the students through their hands-on practice, we did not assess competency in learned skills with the OSCE checklist. Response shift bias could have affected our survey responses. The students' internal frame of reference for the construct being measured—in this case, confidence—changed between the pretest and the posttest due to the influence of the educational activity. Since we did not collect demographic information from the individual participants, we cannot determine if there were any differences between the students who completed and did not complete the postsurvey. In addition to this, we did not include qualitative feedback in our formal evaluation. However, at the end of the academic year, the Community Engaged Physician course directors received both informal and formal feedback noting the naloxone training as a standout session.

Educators aiming to replicate this activity should consider strategic placement within the curriculum. Integrating training on opioid overdose prevention and response requires thoughtful planning to effectively cover the pathophysiology, psychology, prevention, treatment, social determinants, and stigma of SUDs. Given the time and resource demands, educators must identify optimal points within the curriculum for seamless integration. We recommend an incremental integration, by starting with trial sessions during interest group meetings and gradually expanding the program based on feedback and outcomes. This stepwise approach allows for continuous improvement and adaptation. We delivered this training in person. Depending on the institutional resources, the training could be adapted to be delivered virtually. A prior study has suggested that both types of training, in-person and online, result in similar educational outcomes in opioid reversal knowledge and opioid use disorder stigma.[2] Additionally, it is crucial to regularly assess the effectiveness of the training through student feedback and performance evaluations. The use of these data refines the program and ensures it meets educational objectives.

In conclusion, this educational activity demonstrates promising results for the implementation of an interactive opioid response training program to improve preclinical medical students' confidence in effectively identifying and responding to opioid overdoses and adds to medical education curricula on harm reduction. Future activities should focus on incorporating objective measures to assess competency-based skills and long-term evaluation of knowledge retention and skill application during clinical rotations.

Appendices

A. Facilitator Guide.docx
B. Opioid Overdose Statistics Lecture.pptx
C. Harm Reduction Initiatives Lecture.pptx
D. Case-Based Discussion Scenario.pptx

Copyright © 2025 Ovalles Lacruz et al. This is an open-access publication distributed under the terms of the Creative Commons Attribution license.

E. OSCE-Style Checklist.docx

F. Training Session Confidence Survey.docx

All appendices are peer reviewed as integral parts of the Original Publication.

Alexa Ovalles Lacruz: Fourth-Year Medical Student, Florida International University Herbert Wertheim College of Medicine; ORCID: https://orcid.org/0009-0007-5475-6152

Maria S. Valle: Fourth-Year Medical Student, Florida International University Herbert Wertheim College of Medicine

Karla Santoyo: Third-Year Medical Student, Florida International University Herbert Wertheim College of Medicine

Rachel D. Clarke, PhD, CHES: Assistant Professor, Department of Medical Education, and Program Evaluator, Green Family Foundation NeighborhoodHELP, Florida International University Herbert Wertheim College of Medicine

Prasad Bhoite, MBA, MPH, MSc: Data Scientist, Department of Humanities, Health, and Society, Florida International University Herbert Wertheim College of Medicine

Lizzeth N. Alarcon, MD: Assistant Professor, Department of Medical Education, and Co-Course Director, Community Engaged Physician Course, Florida International University Herbert Wertheim College of Medicine

Acknowledgments
We would like to acknowledge the patients with substance use disorder whom this training is ultimately designed to help. We also thank the participants of the training program and the Florida International University Herbert Wertheim College of Medicine community, specifically, Dr. Uchiyama and the Medical Simulation Center team, who have provided continued assistance with this project.

Disclosures
None to report.

Funding/Support
None to report.

Prior Presentations
Ovalles Lacruz A, Valle MS, Santoyo K, Clarke RD, Bhoite P, Alarcon LN. Empowering future physicians: enhancing naloxone competency through early harm reduction training in medical education. Presented at: Annual Dade County Medical Association Research Competition; March 2, 2024; Miami, FL.

Ethical Approval
The Florida International University Institutional Review Board approved this project.

References

1. Drug overdose deaths: facts and figures. National Institute on Drug Abuse. Updated August 2024. Accessed January 22, 2025. https://nida.nih.gov/research-topics/trends-statistics/overdose-death-rates
2. Moses TE, Moreno JL, Greenwald MK, Waineo E. Developing and validating an opioid overdose prevention and response curriculum for undergraduate medical education. *Subst Abus*. 2022;43(1):309-318. https://doi.org/10.1080/08897077.2021.1941515
3. Opioid crisis: addiction, overprescription, and insufficient primary prevention. *Lancet Reg Health Am*. 2023;23:100557. https://doi.org/10.1016/j.lana.2023.100557
4. Opioid overdose. World Health Organization. August 29, 2023. Accessed January 22, 2025. https://www.who.int/news-room/fact-sheets/detail/opioid-overdose
5. Adeosun SO. Stigma by association: to what extent is the attitude toward naloxone affected by the stigma of opioid use disorder? *J Pharm Pract*. 2023;36(4):941-952. https://doi.org/10.1177/08971900221097173
6. Mayet S, Manning V, Williams A, Loaring J, Strang J. Impact of training for healthcare professionals on how to manage an opioid overdose with naloxone: effective, but dissemination is challenging. *Int J Drug Policy*. 2011;22(1):9-15. https://doi.org/10.1016/j.drugpo.2010.09.008
7. Zhang X, Marchand C, Sullivan B, Klass EM, Wagner KD. Naloxone access for emergency medical technicians: an evaluation of a training program in rural communities. *Addict Behav*. 2018;86:79-85. https://doi.org/10.1016/j.addbeh.2018.03.004
8. Goss NC, Haslund-Gourley B, Meredith DM, et al. A comparative analysis of online versus in-person opioid overdose awareness and reversal training for first-year medical students. *Subst Use Misuse*. 2021;56(13):1962-1971. https://doi.org/10.1080/10826084.2021.1958866
9. Riley E, Trotter S, Dayer L, et al. Interprofessional naloxone student training to manage suspected opioid overdose. *Clin Nurse Spec*. 2023;37(1):26-35. https://doi.org/10.1097/NUR.0000000000000720
10. Kwon M, Moody AE, Thigpen J, Gauld A. Implementation of an opioid overdose and naloxone distribution training in a pharmacist laboratory course. *Am J Pharm Educ*. 2020;84(2):7179. https://doi.org/10.5688/ajpe7179
11. Gatewood AK, Van Wert MJ, Andrada AP, Surkan PJ. Academic physicians' and medical students' perceived barriers toward bystander administered naloxone as an overdose prevention strategy. *Addict Behav*. 2016;61:40-46. https://doi.org/10.1016/j.addbeh.2016.05.013
12. Jennings L, Warner T, Bacro-Duverger B. Identification and treatment of opioid withdrawal and opioid use disorder in the

Copyright © 2025 Ovalles Lacruz et al. This is an open-access publication distributed under the terms of the Creative Commons Attribution license.

emergency department. *MedEdPORTAL*. 2020;16:10899. https://doi.org/10.15766/mep_2374-8265.10899

13. Porter R, Barnett J, Blazar M, Pinheiro S, Bowlby L. Addressing the opioid crisis: a dynamic case-based module set for interprofessional educators, learners, and clinicians. *MedEdPORTAL*. 2022;18:11238. https://doi.org/10.15766/mep_2374-8265.11238
14. Taldone S, Lemmon S, Bianco S, et al. Opioid Use Disorder Curriculum: preclerkship pharmacology case-based learning session. *MedEdPORTAL*. 2022;18:11255. https://doi.org/10.15766/mep_2374-8265.11255
15. Fujita AW, LaRosa A, Carter A. Treating withdrawal and pain in inpatients with opioid use disorder: a brief educational intervention for internal medicine residents. *MedEdPORTAL*. 2021;17:11123. https://doi.org/10.15766/mep_2374-8265.11123
16. Findley JC, Schatte D, Power J. A difficult patient encounter: using a standardized patient scenario to teach medical students to treat medication-seeking patients. *MedEdPORTAL*. 2017; 13:10612. https://doi.org/10.15766/mep_2374-8265.10612
17. Patrício MF, Julião M, Fareleira F, Carneiro AV. Is the OSCE a feasible tool to assess competencies in undergraduate medical education? *Med Teach*. 2013;35(6):503-514. https://doi.org/10.3109/0142159X.2013.774330
18. Kong Y. The role of experiential learning on students' motivation and classroom engagement. *Front Psychol*. 2021;12:771272. https://doi.org/10.3389/fpsyg.2021.771272
19. DeVellis RF. *Scale Development: Theory and Applications*. 3rd ed. SAGE; 2012.
20. Mort SC, Díaz SR, Beverly EA. Using contact-based education to destigmatize opioid use disorder among medical students. *Teach Learn Med*. 2021;33(2):196-209. https://doi.org/10.1080/10401334.2020.1820869
21. Oldfield BJ, Tetrault JM, Wilkins KM, Edelman EJ, Capurso NA. Opioid overdose prevention education for medical students: adopting harm reduction into mandatory clerkship curricula. *Subst Abus*. 2020;41(1):29-34. https://doi.org/10.1080/08897077.2019.1621241
22. Smith KR, Shah NK, Adamczyk AL, Weinstein LC, Kelly EL. Harm reduction in undergraduate and graduate medical education: a systematic scoping review. *BMC Med Educ*. 2023;23:986. https://doi.org/10.1186/s12909-023-04931-9

Received: September 15, 2024
Accepted: December 23, 2024
Published: February 14, 2025

Copyright © 2025 Ovalles Lacruz et al. This is an open-access publication distributed under the terms of the Creative Commons Attribution license.
